# Supplementary material for: Tempo and drivers of plant diversification in the European mountain system
Source: Nat Commun. 2022 May 18;13:2750. doi: 10.1038/s41467-022-30394-5 (PMC9117672; doi:10.1038/s41467-022-30394-5)
Supplement: Supplementary file 1 — Supplementary Information [file 41467_2022_30394_MOESM1_ESM.pdf]

Supplementary Information for
Tempo and drivers of plant diversification in the European mountain system

Jan Smyčka, Cristina Roquet, Martí Boleda, Adriana Alberti, Frédéric Boyer, Rolland Douzet, Christophe Perrier, Maxime Rome, Jean-Gabriel Valay, France Denoeud, Kristýna Šemberová, Niklaus E. Zimmermann, Wilfried Thuiller, Patrick Wincker, Inger Greve Alsos, Eric Coissac, *the PhyloAlps consortium*, Sébastien Lavergne

Corresponding author: Jan Smyčka smyckaj@gmail.com

This PDF includes:

Supplementary Figure 1 – Evolutionary assembly across 5 major mountain regions in Europe…………….2
Supplementary Figure 2 – Current and equilibrium species proportions across ecological gradients………3
Supplementary Figure 3 – Ancestral reconstructions of bedrock and elevational niches……………………4
Supplementary Methods……………………………..………………………………………………….9
1 Molecular dating calibrations………………………………………….……………………….9
2 Multi-clade time-dependent diversification model R vignette……..…….………………..…..10
3 Sensitivity of temperature-dependent diversification models…………………..…………..22
4 Multi-clade state-dependent diversification model R vignette…….……………………….…25
5 Compilation of geographic information……………..………………….………………….....39
6 Species sampling and taxonomic treatment……….…………………………………..….…..40
Supplementary Note – The PhyloAlps consortium…………………………………………………..42
Supplementary References……………………………………………………………………………43

Supplementary Figure 1: Rates of (a) constant-state speciation across 5 regions of European mountain system, (b) common migration rates between each pair of regions and (c) common extinction rates. The dots represent mean parameter estimates and the bars indicate 95% credibility intervals, based on 5000 MCMC samples from the model posterior. Black dots and bars represent the shared parameter estimates from the multi-clade model, where each of the 6 phylogenies is regarded as an independent realization of the same diversification process. The estimate is based on ClaSSE model with the best AIC (no state-change speciation, region-specific constant-state speciation rates, single extinction rate, single migration rate). Source data are provided as a Source Data file.

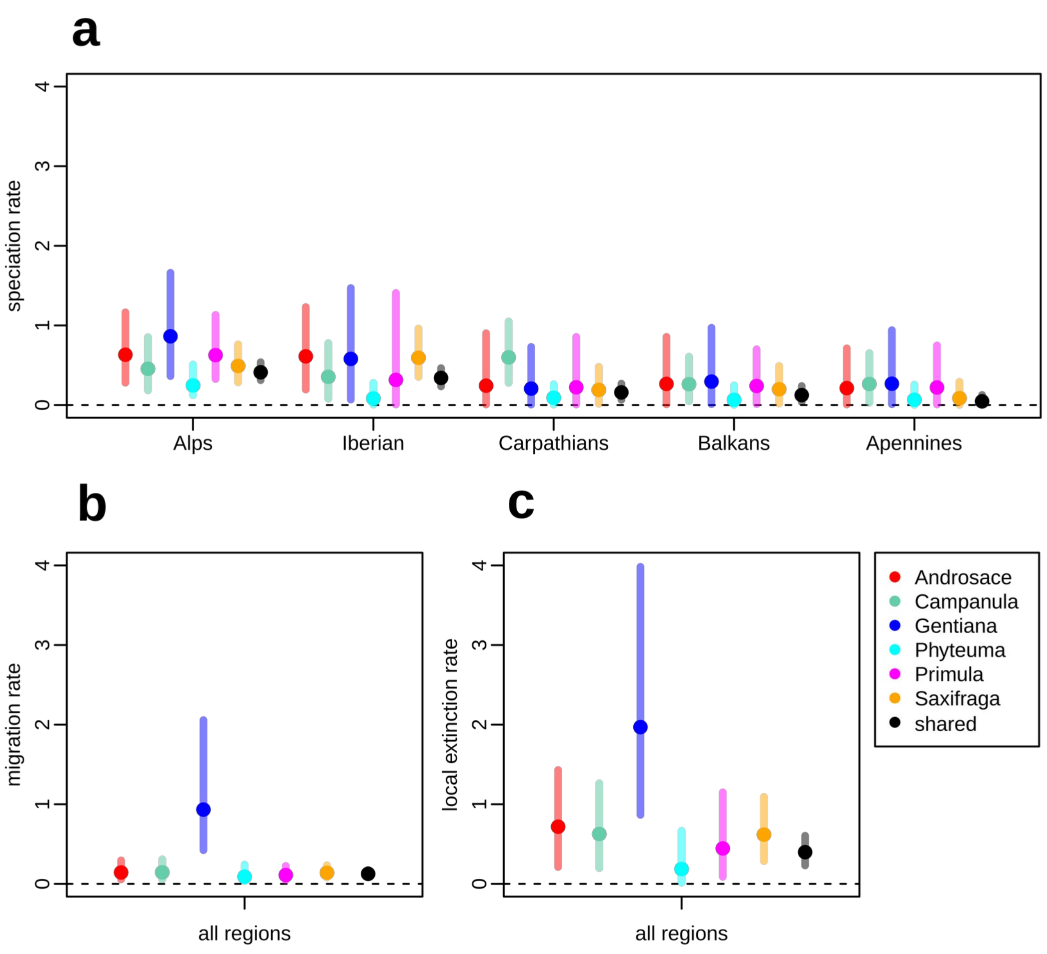

Supplementary Figure 2: Current and stationary proportions of (a) calcareous specialists, siliceous specialists or bedrock generalist species; and (b) high elevation specialists, mid-elevation specialists, and elevation generalist species. The color scheme follows the same one as in Supplementary Figure 1, but includes shading to differentiate niche states. Stationary proportions represent a limit distribution of states of the model for time going to infinity, and are calculated by eigendecomposition of ClaSSE model matrices constructed from mean posterior parameter estimates, with the function *stationary.freq.classe* in R package *diversitree*. Source data are provided as a Source Data file.

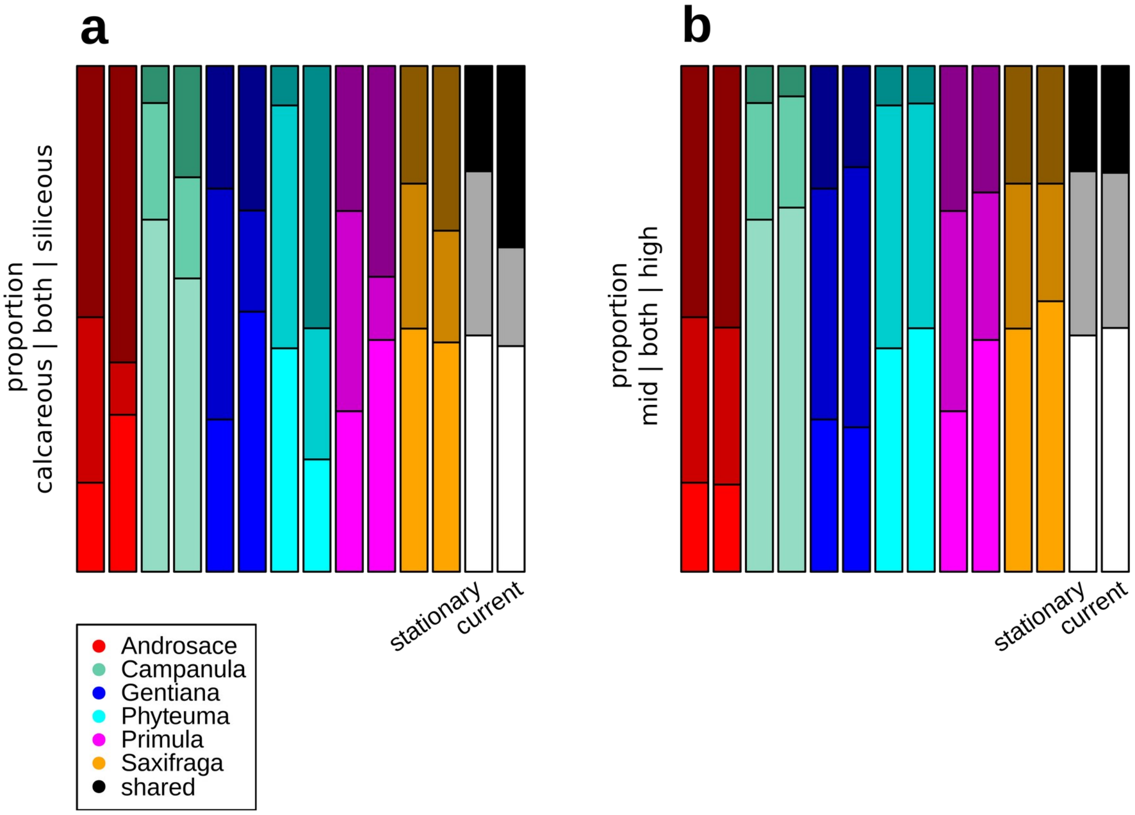

Supplementary Figure 3: Ancestral state estimation of (a) bedrock and (b) elevation niches for the six lineages, based on median posterior parameter estimates from respective ClaSSE models using the marginal reconstruction algorithm provided in the R package *HiSSE*. The bar at the bottom of each phylogeny represents a time interval of 1 Ma.

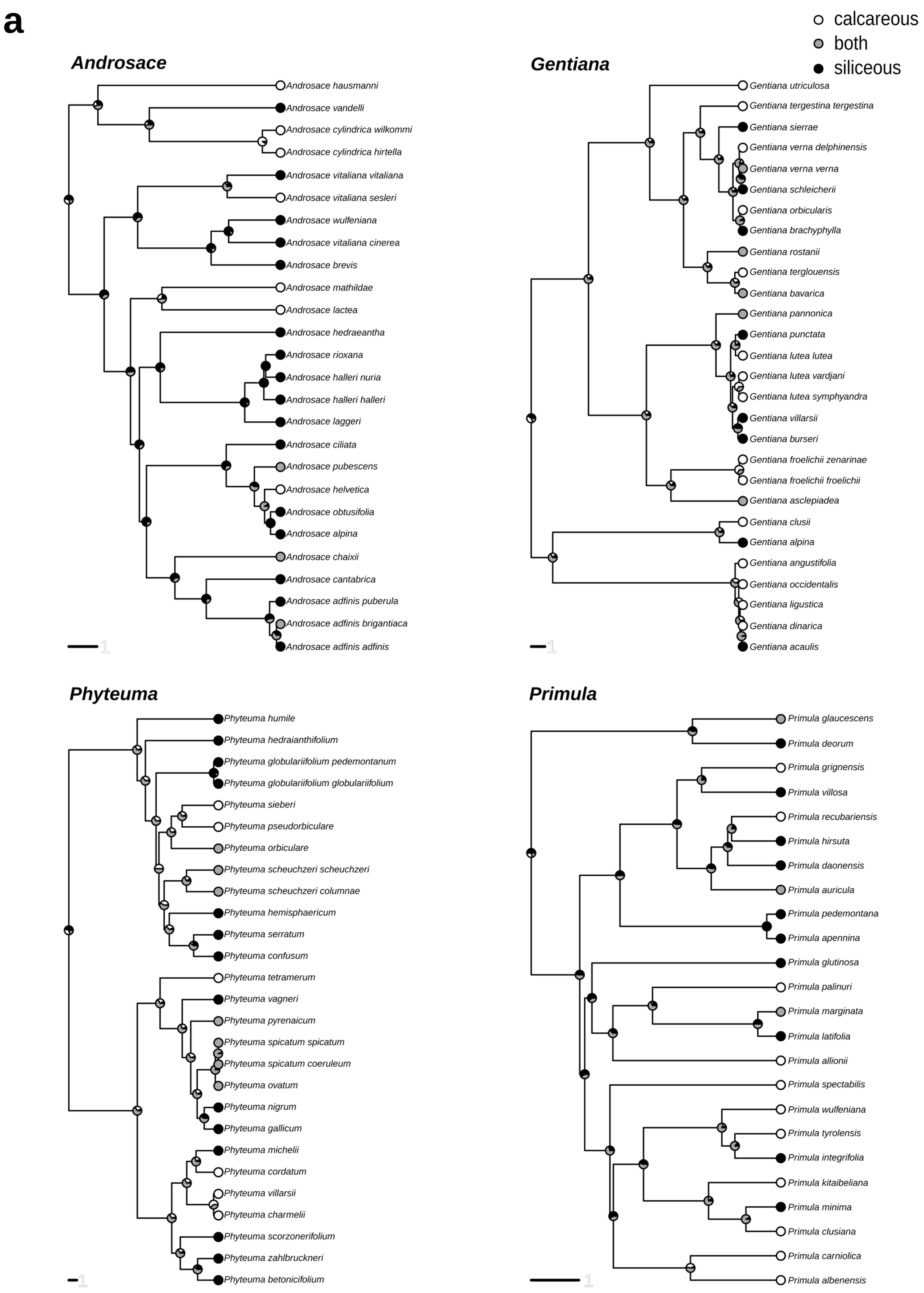
a
calcareous
both
siliceous
Androsace
Androsace hausmanni
Androsace vandelli
Androsace cylindrica wilkommi
Androsace cylindrica hirtella
Androsace vitaliana vitaliana
Androsace vitaliana sesleri
Androsace wulfeniana
Androsace vitaliana cinerea
Androsace brevis
Androsace mathildae
Androsace lactea
Androsace hedraeantha
Androsace rioxana
Androsace halleri nuria
Androsace halleri halleri
Androsace laggeri
Androsace ciliata
Androsace pubescens
Androsace helvetica
Androsace obtusifolia
Androsace alpina
Androsace chaixii
Androsace cantabrica
Androsace adfinis puberula
Androsace adfinis brigantiaca
Androsace adfinis adfinis
1
Gentiana
Gentiana utriculosa
Gentiana tergestina tergestina
Gentiana sierrae
Gentiana verna delphinensis
Gentiana verna verna
Gentiana schleicherii
Gentiana orbicularis
Gentiana brachyphylla
Gentiana rostanii
Gentiana terglouensis
Gentiana bavarica
Gentiana pannonica
Gentiana punctata
Gentiana lutea lutea
Gentiana lutea vardjani
Gentiana lutea symphyandra
Gentiana villarsii
Gentiana burseri
Gentiana froelichii zenarinae
Gentiana froelichii froelichii
Gentiana asclepiadea
Gentiana clusii
Gentiana alpina
Gentiana angustifolia
Gentiana occidentalis
Gentiana ligustica
Gentiana dinarica
Gentiana acaulis
1
Phyteuma
Phyteuma humile
Phyteuma hedraianthifolium
Phyteuma globulariifolium pedemontanum
Phyteuma globulariifolium globulariifolium
Phyteuma sieberi
Phyteuma pseudorbiculare
Phyteuma orbiculare
Phyteuma scheuchzeri scheuchzeri
Phyteuma scheuchzeri columnae
Phyteuma hemisphaericum
Phyteuma serratum
Phyteuma confusum
Phyteuma tetramerum
Phyteuma vagneri
Phyteuma pyrenaicum
Phyteuma spicatum spicatum
Phyteuma spicatum coeruleum
Phyteuma ovatum
Phyteuma nigrum
Phyteuma gallicum
Phyteuma michelii
Phyteuma cordatum
Phyteuma villarsii
Phyteuma charmelii
Phyteuma scorzonerifolium
Phyteuma zahlbruckneri
Phyteuma betonicifolium
1
Primula
Primula glaucescens
Primula deorum
Primula grignensis
Primula villosa
Primula recubariensis
Primula hirsuta
Primula daonensis
Primula auricula
Primula pedemontana
Primula apennina
Primula glutinosa
Primula palinuri
Primula marginata
Primula latifolia
Primula allionii
Primula spectabilis
Primula wulfeniana
Primula tyrolensis
Primula integrifolia
Primula kitaibeliana
Primula minima
Primula clusiana
Primula carniolica
Primula albenensis
1

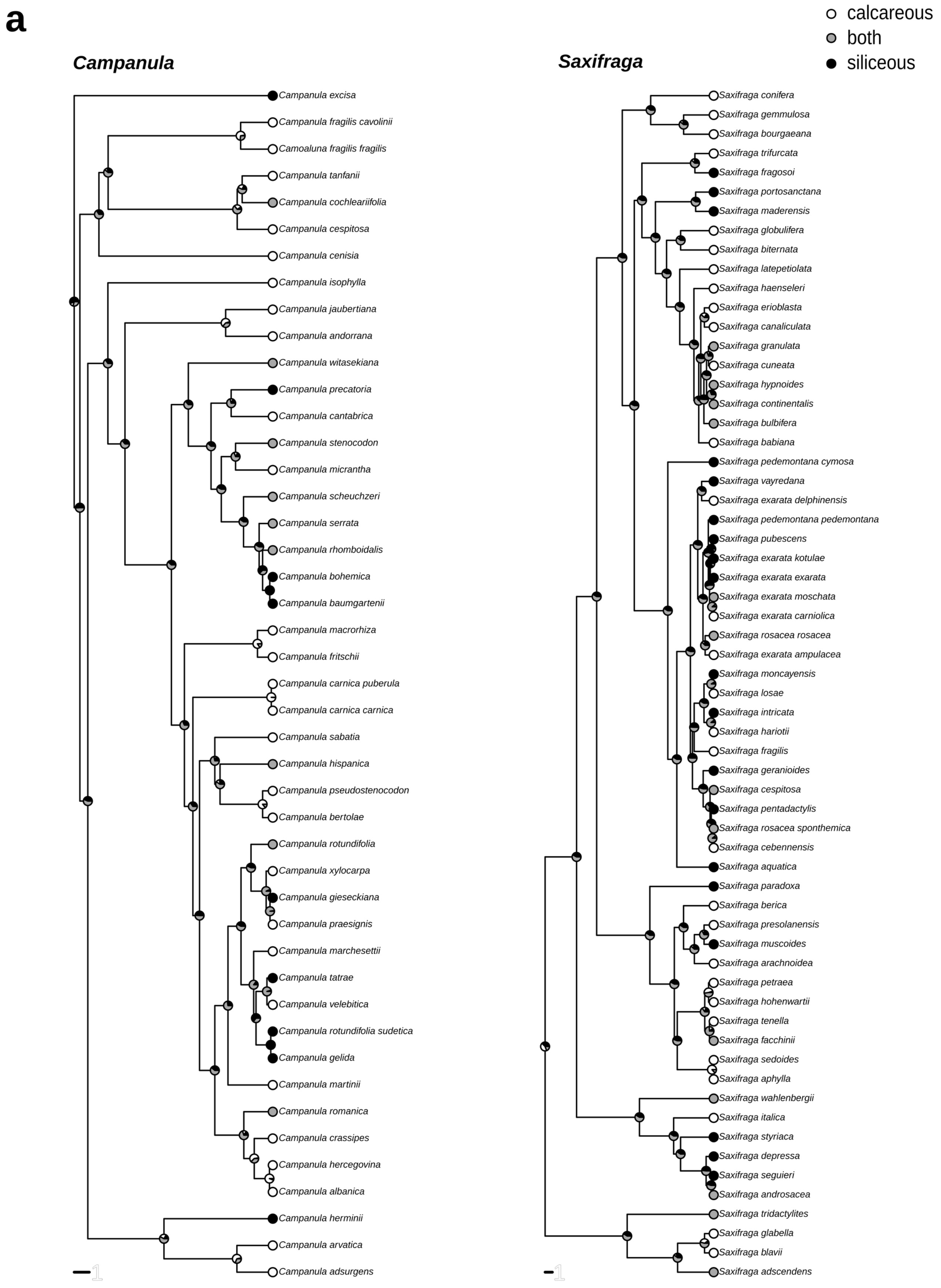

a
calcareous
both
siliceous
Campanula
Campanula excisa
Campanula fragilis cavolinii
Camoaluna fragilis fragilis
Campanula tanfanii
Campanula cochleariifolia
Campanula cespitosa
Campanula cenisia
Campanula isophylla
Campanula jaubertiana
Campanula andorrana
Campanula witasekiana
Campanula precatoria
Campanula cantabrica
Campanula stenocodon
Campanula micrantha
Campanula scheuchzeri
Campanula serrata
Campanula rhomboidalis
Campanula bohemica
Campanula baumgartenii
Campanula macrorhiza
Campanula fritschii
Campanula carnica puberula
Campanula carnica carnica
Campanula sabatia
Campanula hispanica
Campanula pseudostenocodon
Campanula bertolae
Campanula rotundifolia
Campanula xylocarpa
Campanula gieseckiana
Campanula praesignis
Campanula marchesettii
Campanula tatrae
Campanula velebitica
Campanula rotundifolia sudetica
Campanula gelida
Campanula martinii
Campanula romanica
Campanula crassipes
Campanula hercegovina
Campanula albanica
Campanula herminii
Campanula arvatica
Campanula adsurgens
1
Saxifraga
Saxifraga conifera
Saxifraga gemmulosa
Saxifraga bourgaeana
Saxifraga trifurcata
Saxifraga fragosoi
Saxifraga portosanctana
Saxifraga maderensis
Saxifraga globulifera
Saxifraga biternata
Saxifraga latepetiolata
Saxifraga haenseleri
Saxifraga erioblasta
Saxifraga canaliculata
Saxifraga granulata
Saxifraga cuneata
Saxifraga hypnoides
Saxifraga continentalis
Saxifraga bulbifera
Saxifraga babiana
Saxifraga pedemontana cymosa
Saxifraga vayredana
Saxifraga exarata delphinensis
Saxifraga pedemontana pedemontana
Saxifraga pubescens
Saxifraga exarata kotulae
Saxifraga exarata exarata
Saxifraga exarata moschata
Saxifraga exarata carniolica
Saxifraga rosacea rosacea
Saxifraga exarata ampulacea
Saxifraga moncayensis
Saxifraga losae
Saxifraga intricata
Saxifraga hariotii
Saxifraga fragilis
Saxifraga geranioides
Saxifraga cespitosa
Saxifraga pentadactylis
Saxifraga rosacea sponthemica
Saxifraga cebennensis
Saxifraga aquatica
Saxifraga paradoxa
Saxifraga berica
Saxifraga presolanensis
Saxifraga muscoides
Saxifraga arachnoidea
Saxifraga petraea
Saxifraga hohenwartii
Saxifraga tenella
Saxifraga facchinii
Saxifraga sedoides
Saxifraga aphylla
Saxifraga wahlenbergii
Saxifraga italica
Saxifraga styriaca
Saxifraga depressa
Saxifraga seguieri
Saxifraga androsacea
Saxifraga tridactylites
Saxifraga glabella
Saxifraga blavii
Saxifraga adscendens
1

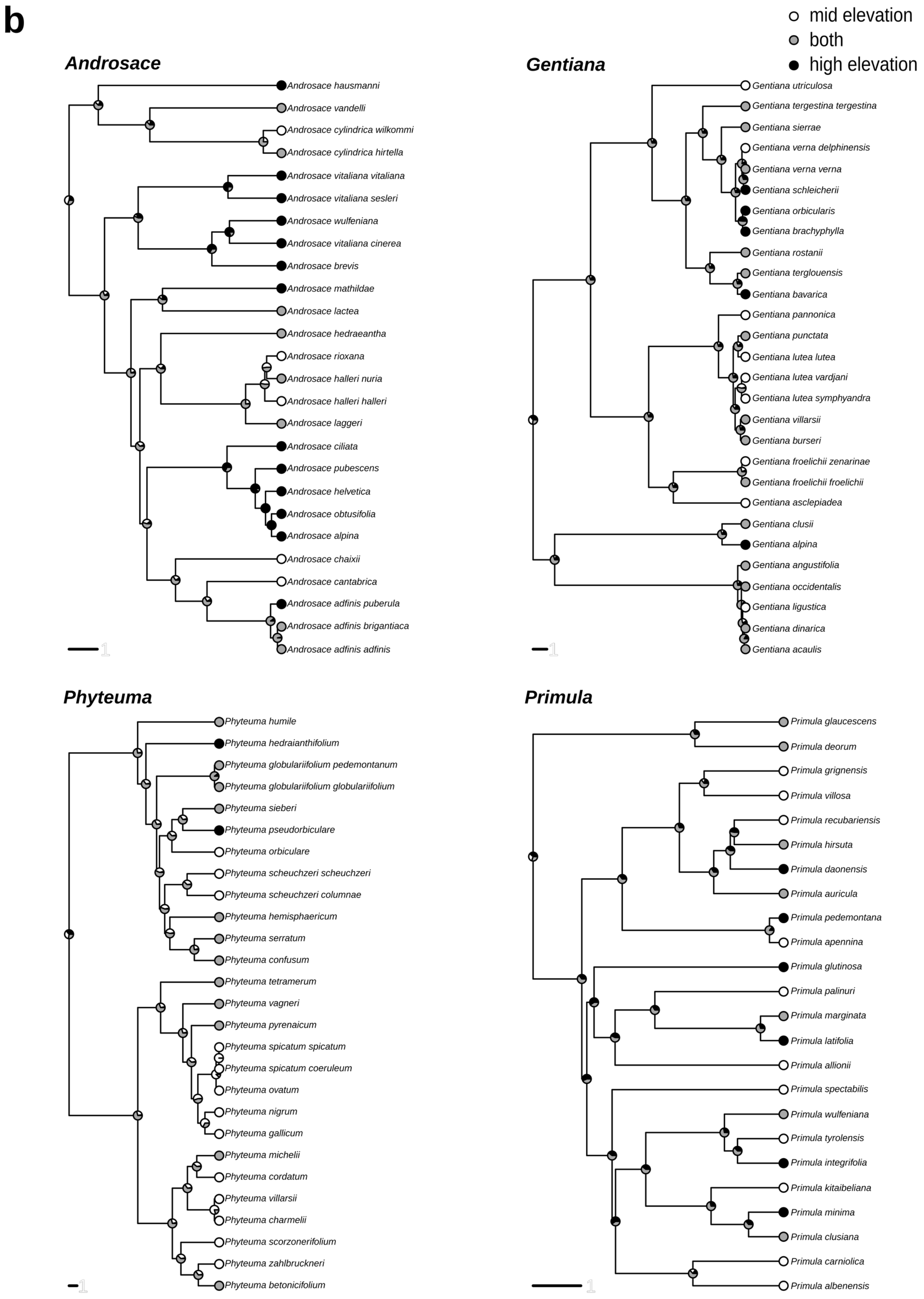
b
mid elevation
both
high elevation
Androsace
Androsace hausmanni
Androsace vandelli
Androsace cylindrica wilkommi
Androsace cylindrica hirtella
Androsace vitaliana vitaliana
Androsace vitaliana sesleri
Androsace wulfeniana
Androsace vitaliana cinerea
Androsace brevis
Androsace mathildae
Androsace lactea
Androsace hedraeantha
Androsace rioxana
Androsace halleri nuria
Androsace halleri halleri
Androsace laggeri
Androsace ciliata
Androsace pubescens
Androsace helvetica
Androsace obtusifolia
Androsace alpina
Androsace chaixii
Androsace cantabrica
Androsace adfinis puberula
Androsace adfinis brigantiaca
Androsace adfinis adfinis
1
Gentiana
Gentiana utriculosa
Gentiana tergestina tergestina
Gentiana sierrae
Gentiana verna delphinensis
Gentiana verna verna
Gentiana schleicherii
Gentiana orbicularis
Gentiana brachyphylla
Gentiana rostanii
Gentiana terglouensis
Gentiana bavarica
Gentiana pannonica
Gentiana punctata
Gentiana lutea lutea
Gentiana lutea vardjani
Gentiana lutea symphyandra
Gentiana villarsii
Gentiana burseri
Gentiana froelichii zenarinae
Gentiana froelichii froelichii
Gentiana asclepiadea
Gentiana clusii
Gentiana alpina
Gentiana angustifolia
Gentiana occidentalis
Gentiana ligustica
Gentiana dinarica
Gentiana acaulis
1
Phyteuma
Phyteuma humile
Phyteuma hedraianthifolium
Phyteuma globulariifolium pedemontanum
Phyteuma globulariifolium globulariifolium
Phyteuma sieberi
Phyteuma pseudorbiculare
Phyteuma orbiculare
Phyteuma scheuchzeri scheuchzeri
Phyteuma scheuchzeri columnae
Phyteuma hemisphaericum
Phyteuma serratum
Phyteuma confusum
Phyteuma tetramerum
Phyteuma vagneri
Phyteuma pyrenaicum
Phyteuma spicatum spicatum
Phyteuma spicatum coeruleum
Phyteuma ovatum
Phyteuma nigrum
Phyteuma gallicum
Phyteuma michelii
Phyteuma cordatum
Phyteuma villarsii
Phyteuma charmelii
Phyteuma scorzonerifolium
Phyteuma zahlbruckneri
Phyteuma betonicifolium
1
Primula
Primula glaucescens
Primula deorum
Primula grignensis
Primula villosa
Primula recubariensis
Primula hirsuta
Primula daonensis
Primula auricula
Primula pedemontana
Primula apennina
Primula glutinosa
Primula palinuri
Primula marginata
Primula latifolia
Primula allionii
Primula spectabilis
Primula wulfeniana
Primula tyrolensis
Primula integrifolia
Primula kitaibeliana
Primula minima
Primula clusiana
Primula carniolica
Primula albenensis
1

b

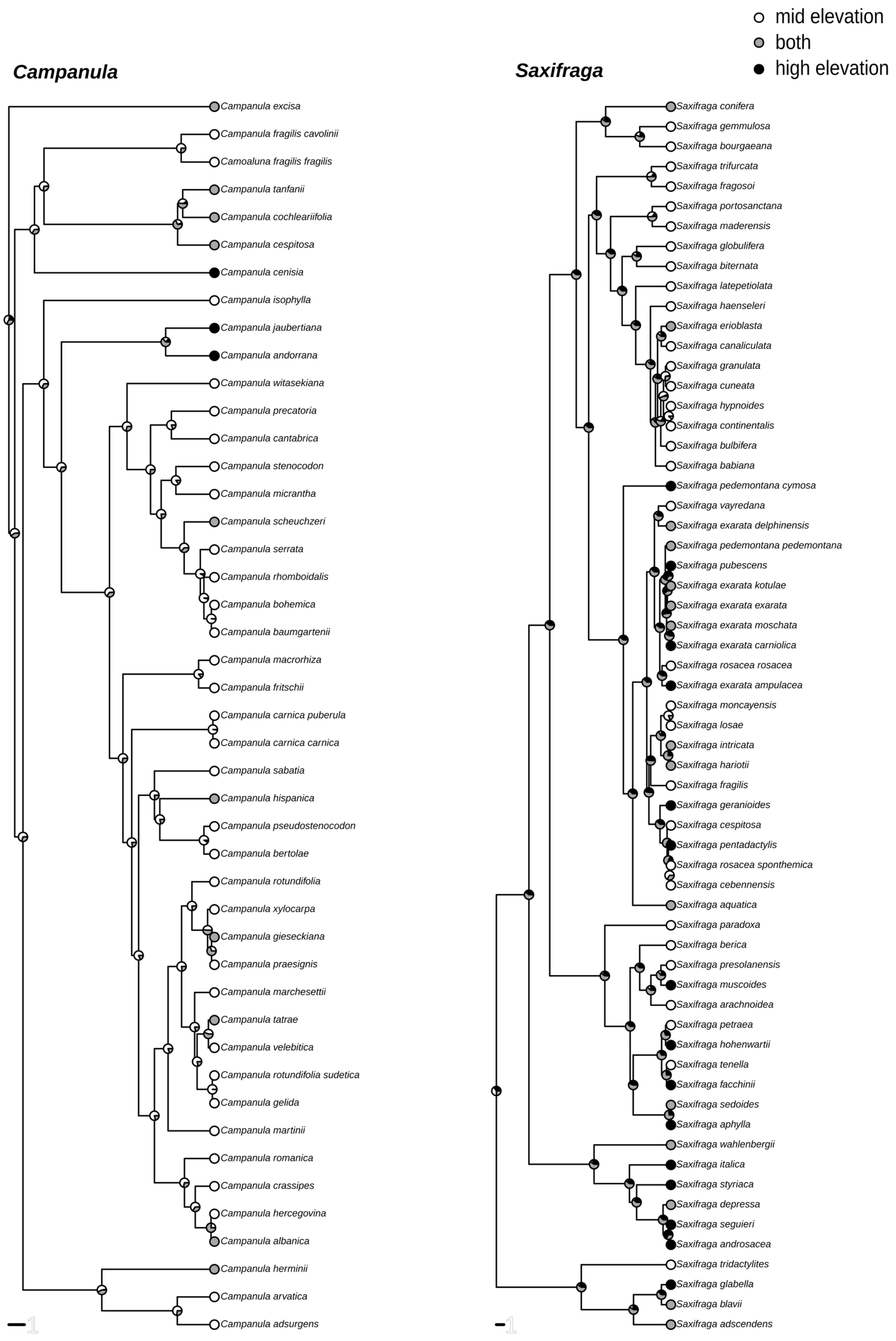
mid elevation
both
high elevation
Campanula
Campanula excisa
Campanula fragilis cavolinii
Camoaluna fragilis fragilis
Campanula tanfanii
Campanula cochleariifolia
Campanula cespitosa
Campanula cenisia
Campanula isophylla
Campanula jaubertiana
Campanula andorrana
Campanula witasekiana
Campanula precatoria
Campanula cantabrica
Campanula stenocodon
Campanula micrantha
Campanula scheuchzeri
Campanula serrata
Campanula rhomboidalis
Campanula bohemica
Campanula baumgartenii
Campanula macrorhiza
Campanula fritschii
Campanula carnica puberula
Campanula carnica carnica
Campanula sabatia
Campanula hispanica
Campanula pseudostenocodon
Campanula bertolae
Campanula rotundifolia
Campanula xylocarpa
Campanula gieseckiana
Campanula praesignis
Campanula marchesettii
Campanula tatrae
Campanula velebitica
Campanula rotundifolia sudetica
Campanula gelida
Campanula martinii
Campanula romanica
Campanula crassipes
Campanula hercegovina
Campanula albanica
Campanula herminii
Campanula arvatica
Campanula adsurgens
1
Saxifraga
Saxifraga conifera
Saxifraga gemmulosa
Saxifraga bourgaeana
Saxifraga trifurcata
Saxifraga fragosoi
Saxifraga portosanctana
Saxifraga maderensis
Saxifraga globulifera
Saxifraga biternata
Saxifraga latepetiolata
Saxifraga haenseleri
Saxifraga erioblasta
Saxifraga canaliculata
Saxifraga granulata
Saxifraga cuneata
Saxifraga hypnoides
Saxifraga continentalis
Saxifraga bulbifera
Saxifraga babiana
Saxifraga pedemontana cymosa
Saxifraga vayredana
Saxifraga exarata delphinensis
Saxifraga pedemontana pedemontana
Saxifraga pubescens
Saxifraga exarata kotulae
Saxifraga exarata exarata
Saxifraga exarata moschata
Saxifraga exarata carniolica
Saxifraga rosacea rosacea
Saxifraga exarata ampulacea
Saxifraga moncayensis
Saxifraga losae
Saxifraga intricata
Saxifraga hariotii
Saxifraga fragilis
Saxifraga geranioides
Saxifraga cespitosa
Saxifraga pentadactylis
Saxifraga rosacea sponthemica
Saxifraga cebennensis
Saxifraga aquatica
Saxifraga paradoxa
Saxifraga berica
Saxifraga presolanensis
Saxifraga muscoides
Saxifraga arachnoidea
Saxifraga petraea
Saxifraga hohenwartii
Saxifraga tenella
Saxifraga facchinii
Saxifraga sedoides
Saxifraga aphylla
Saxifraga wahlenbergii
Saxifraga italica
Saxifraga styriaca
Saxifraga depressa
Saxifraga seguieri
Saxifraga androsacea
Saxifraga tridactylites
Saxifraga glabella
Saxifraga blavii
Saxifraga adscendens
1

Supplementary Methods

1 Molecular dating calibrations

For Campanulaceae, in accordance with Mansion et al. 2011[1], we defined the following calibration bounds:

- crown age of *C. carpatica*+*C. pulla*+*C.pyramidalis* to 16.5-56 Ma BP. The minimum bound is based on fossil seed of *C. palaeopyramidalis*[2]. The maximum bound is based on inferred split between *Codonopsis* and *Campanula*[3].
- root of Campanulaceae to 16.5-56 Ma BP.

For Gentianaceae , in accordance with Favre et al. 2016[4], we defined the following bounds:

- crown age of *Gentiana*+*Gentianopsis*+*Lomatogonium*+*Swertia* to 5-100 Ma BP. The minimum bound is based on fossil seeds of *Gentiana*[5]. The maximum bound is based on the conservative estimate from Rybzcinski et al. 2014[6].
- root of Gentianaceae to 33.6-100 Ma BP. The minimum bound is based on fossil pollen of *Lisianthus*[7].

Alternatively, the the fossil seed of *Gentiana*[5] may be attributed specifically to *G. cruciata* section, and the respective prior would thus refer to crown age of *Gentiana cruciata*+*Gentiana pneumonanthe*. Similarly, the root prior may alternatively be restricted to 40-50 Ma BP, based on estimate for this divergence event from Merckx et al. 2013[8]. These changes in dating calibration result in very similar maximum credibility dates within the ingroup with slightly lower uncertainty, e.g. the crown age of the three ingroup *Gentiana* clades would be 14.3 Ma (95% CI 8.9-20.7 Ma) instead of 15.4 Ma (95% CI 6.4-28.5 Ma). For the used and the alternative maximum credibility phylogeny of *Gentiana*, see Supplementary Software 1 and 2, respectively.

For Primulaceae , in accordance with Xing and Ree 2017[9], we defined the following bounds:

- crown age of *Cortusa*+*Hottonia*+*Primula*+*Soldanella* to 15.97-72 Ma BP. The minimum bound is based on fossil seeds of *Primula rosiae*[10]. The maximum bound is based on primuloid fossil flower that cannot be attributed directly to Primulaceae[11].
- crown age of *Androsace*+*Cortusa*+*Douglasia*+*Hottonia*+*Primula*+*Soldanella* to 5.3-72 Ma BP. The minimum bound is based on fossil seeds of *Androsace*[12].
- crown age of *Anagallis*+*Androsace*+*Cortusa*+*Douglasia*+*Hottonia*+*Lysimachia*+*Primula*+ *Soldanella*+*Trientalis* to 28-72 Ma BP. The minimum bound is based on fossil seed attributable to Primuloideae.[13].
- root of Primulaceae to 48.6-72 Ma BP. The minimum bound is based on fossils of *Ardisia*[14].

Alternatively, the fossil seed of Primuloideae[13] could be attributed to *Lysimachia* as in Boucher 2016[15], and the respective prior would thus refer to the crown age of *Lysimachia*+*Cyclamen.* This change in calibration results in very similar dates within the ingroups, e.g. crown age of *Primula* sect. *Auriculata* 5.1 Ma (95% CI 3.0-7.5 Ma) instead of 5.2 Ma (95% CI 3.2-7.9 Ma) and crown age of *Androsace* sect. *Aretia* 7.3 Ma (95% CI 4.5-10.9 Ma) instead of 7.5 Ma (95% CI 4.7-10.7 Ma). For the used and the alternative maximum credibility phylogeny of *Primula* and *Androsace*, see Supplementary Software 1 and 2, respectively.

For Saxifragaceae+Grossulariaceae, we restricted, in accordance with Ebersbach et al. 2017[16] the following bounds:

- crown age of *Ribes* to 14.5-125 Ma BP. The minimum age is based on fossil leaves of *Ribes webbii* attributable to *Ribes* group *Calobotrya*[17]. The maximum age is based on earliest angiosperm fossil evidence[18].
- root of Saxifragaceae+Grossulariaceae to 42-125 Ma BP. Minimum age is based on fossil leaves of *Ribes axelrodii*[17].

Alternatively, the root age of Saxifragaceae+Grossulariaceae could be secondary-calibrated by maximum credibility interval from Ebersbach et al. 2017[16], who have more extensive taxon sampling than our study, to 74-93 Ma BP. Such strategy would result in slightly older maximum credibility dates of the ingroup with lower uncertainty, e.g. crown age of *Saxifraga* sect. *Saxifraga* 27.2 Ma (95% CI 18.9-36.1 Ma) instead of 23.2 Ma (95% CI 12.5-38.1 Ma). For the used and the alternative maximum credibility phylogeny of *Saxifraga*, see see Supplementary Software 1 and 2, respectively.

2 Multi-clade time-dependent diversification model R vignette

This vignette aims to demonstrate the multi-clade time-dependent diversification model used in the paper. The main idea behind this method is that the analyzed phylogenies are *n* observations of shared diversification process and the likelihood of observing them together is thus a product of individual likelihood functions of each phylogeny (Supplementary Equation 1).

$$\mathrm{L}_{\mathrm{alltogether}}(x)=\prod_{i=1}^{n} \mathrm{L}_i(x)$$

(Supplementary Equation 1)

This product likelihood function can be searched for optimal estimate of diversification parameters shared by all the phylogenies. On a technical side, this approach is implemented by modifying the likelihood expressions in the functions *fit_bd* and *fit_env* of the *RPANDA* package[19] in R.

Here we use simulated phylogenies from time-constant birth-death process and a birth-death process with speciation rate dependent on past temperatures to test whether the parameter estimates of single lineage and shared diversification models match those of the generating process. Apart from that, we also explore whether the AIC comparison is useful approach to check if the shared model fits the data better than a set of single-lineage models. Our results show that the parameters of both shared and single-lineage diversification models as we used them throughout the paper can be unambiguously identified, addressing thus the recent criticism of diversification models[20].

Birth-death model

Here we generate a set of 6 phylogenetic trees using a birth death model with constant diversification rates reflecting those detected in the mountain plants dataset (lambda=0.4019096 and mu=0.2594446) over 10 Ma.

```
library(RPANDA)
## Loading required package: picante
## Loading required package: ape
## Loading required package: vegan
## Loading required package: permute
## Loading required package: lattice
## This is vegan 2.5-6
## Loading required package: nlme
library(geiger)
library(pspline)

lambdabd=0.4019096
mubd=0.2594446

rbdtreesim=function(lambdabd,mubd){rbdtree(lambdabd,mubd,10)}
tbdlist=mapply(rbdtreesim, rep(lambdabd,6),rep(mubd,6),SIMPLIFY = F)
```

Then, we define a function for estimating shared parameters of birth-death process across multiple phylogenies. The function is a modification of *fit_bd* from *RPANDA*, the critical part with the product likelihood function is found between the # lines.

```
fit_bdmulti=function (phylolist, tot_timelist, f.lamb, f.mu, lamb_par, mu_par, 
flist,
          meth = "Nelder-Mead", cst.lamb = FALSE, cst.mu = FALSE, expo.lamb = 
FALSE,
          expo.mu = FALSE, fix.mu = FALSE, dt = 0, cond = "crown")
{
  #the calculation of total n for aicc
  ntiplist=lapply(phylolist,Ntip)
  nobs=Reduce("+",ntiplist)

  if (fix.mu == FALSE) {
    init <- c(lamb_par, mu_par)
    p <- length(init)
    optimLH <- function(init) {
      lamb_par <- init[1:length(lamb_par)]
      mu_par <- init[(1 + length(lamb_par)):length(init)]
      f.lamb.par <- function(t) {
        abs(f.lamb(t, lamb_par))
      }
      f.mu.par <- function(t) {
        abs(f.mu(t, mu_par))
      }

################################################################################
#####
      #the LH is now a sum of likelihoods for different trees, each tree can 
have a
      #different total age (tot_time) and sampling proportion (f)
      lhsinpar=function(phylo, tot_time, f){
        likelihood_bd(phylo, tot_time, f.lamb.par,
                      f.mu.par, f, cst.lamb = cst.lamb, cst.mu = cst.mu,
                      expo.lamb = expo.lamb, expo.mu = expo.mu, dt = dt,
                      cond = cond)
        }
      likelihoodlist=mapply(lhsinpar, phylolist, tot_timelist, flist)
      LH=Reduce("+",likelihoodlist)

      return(-LH)

################################################################################
#####
    }
    temp <- suppressWarnings(optim(init, optimLH, method = meth))
    lamb.par <- temp$par[1:length(lamb_par)]
    mu.par <- temp$par[(1 + length(lamb_par)):length(init)]
    f.lamb.par <- function(t) {
      f.lamb(t, lamb.par)
    }
    f.mu.par <- function(t) {
      f.mu(t, mu.par)
    }
    res <- list(model = "birth death", LH = -temp$value,
                aicc = 2 * temp$value + 2 * p + (2 * p * (p + 1))
                /(nobs -  p - 1), lamb_par = lamb.par, mu_par = mu.par,
                f.lamb = Vectorize(f.lamb.par), f.mu = Vectorize(f.mu.par))
```

```
  }
  else {
    init <- c(lamb_par)
    p <- length(init)
    optimLH <- function(init) {
      lamb_par <- init[1:length(lamb_par)]
      f.lamb.par <- function(t) {
        abs(f.lamb(t, lamb_par))
      }
      f.mu.par <- function(t) {
        abs(f.mu(t, mu_par))
      }

################################################################################
#####
      #the LH is now a sum of likelihoods for different trees, each tree can
have a
      #different total age (tot_time) and sampling proportion (f)
      lhsinpar=function(phylo, tot_time, f){
        likelihood_bd(phylo, tot_time, f.lamb.par,
                      f.mu.par, f, cst.lamb = cst.lamb, cst.mu = cst.mu,
                      expo.lamb = expo.lamb, expo.mu = expo.mu, dt = dt,
                      cond = cond)
        }
      likelihoodlist=mapply(lhsinpar, phylolist, tot_timelist, flist)
      LH=Reduce("+",likelihoodlist)

      return(-LH)

################################################################################
#####
    }
    temp <- suppressWarnings(optim(init, optimLH, method = meth))
    lamb.par <- temp$par[1:length(lamb_par)]
    f.lamb.par <- function(t) {
      f.lamb(t, lamb.par)
    }
    f.mu.par <- function(t) {
      f.mu(t, mu_par)
    }
    res <- list(model = "birth.death", LH = -temp$value,
                aicc = 2 * temp$value + 2 * p + (2 * p * (p + 1))
                /(nobs - p - 1), lamb_par = lamb.par, f.lamb =
Vectorize(f.lamb.par))
  }
  class(res) <- "fit.bd"
  return(res)
}
```

In the next step, we fit the model with shared parameters across the phylogenies.

```
#get total time for each tree
tot_timebdfn=function(phylo){max(node.age(phylo)$ages)}
tot_timebdlist=lapply(tbdlist,tot_timebdfn)

#list of sampling proportion
fbdlist=as.list(rep(1,6))

#starting points for ML search
lamb_parbd<-c(runif(1,0,1))
mu_parbd<-c(runif(1,0,lamb_parbd[1]/2))

#functions of time dependence (constant)
f.lambbd <-function(t,y){y[1]}
f.mubd<-function(t,y){y[1]}

#fit
multibd=fit_bdmulti(tbdlist, tot_timebdlist,
                  f.lamb=f.lambbd,f.mu=f.mubd,
                  lamb_par=lamb_parbd,mu_par=mu_parbd,
                  cst.lamb =T,cst.mu = T,
                  fbdlist, dt=1e-3)

multilambbd=abs(multibd$lamb_par)
multimubd=abs(multibd$mu_par)
multiLHbd=multibd$LH
```

For comparison, we then fit the diversification model on each tree separately.

```
# fit
ferbd=function(phylo, tot_time){
    fit_bd(phylo,tot_time,
           f.lamb=f.lambbd,f.mu=f.mubd,
           lamb_par=lamb_parbd,mu_par=mu_parbd,
           cst.lamb = T,cst.mu = T,
           f=1, dt=1e-3)
  }
singlebd=mapply(ferbd,tbdlist,tot_timebdlist)


#vectorize model characteristics from a list
singlelambbd=rep(0,6)
singlemubd=rep(0,6)
singleLHbd=rep(0,6)

for(i in 1:6){
  tempres=singlebd[,i]
  singlelambbd[i]=abs(tempres$lamb_par[1])
  singlemubd[i]=abs(tempres$mu_par[1])
  singleLHbd[i]=tempres$LH
}
```

The plot showing the parameter estimates reflects that shared estimates of speciation and extinction rates closely match the original values, with single-tree estimates being scattered around with larger variance. The aggregation of estimates around the original values suggests that both shared and single-tree model are identifiable from the generated data.

```
plot(c(singlelambbd,singlemubd)~c(rep(1,6),rep(2,6)),
     xlim=c(0.5,2.5),ylim=c(0,1.2),xlab="",ylab="parameter estimate", pch=20,
xaxt="n")
axis(1, at = c(1,2), labels = c("lambda", "mu"))
points(1,multilambbd, col="red", pch=19)
points(2,multimubd, col="red", pch=19)
points(1,lambdabd, pch=4,cex=2)
points(2,mubd, pch=4,cex=2)
legend(x='topright', legend=c("single-tree estimates", "shared estimate", "real
value"), col=c("black", "red", "black"), pch=c(20,19,4))
```

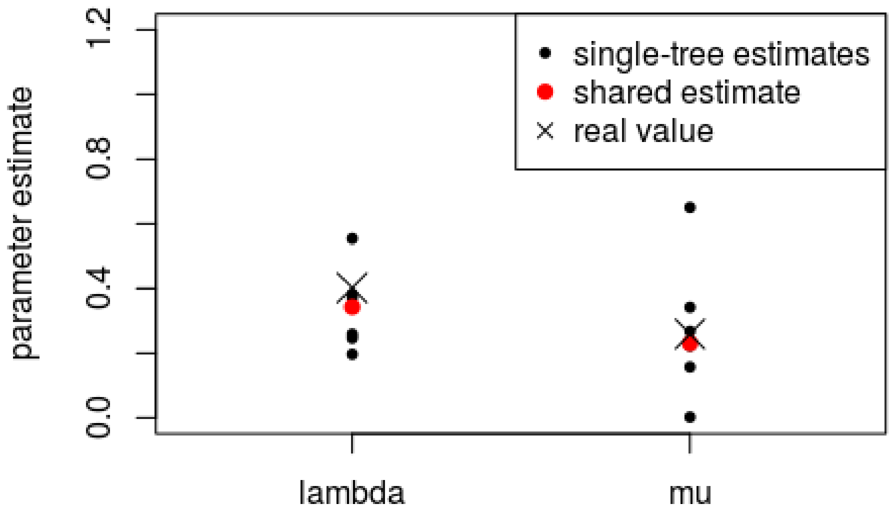

Supplementary Figure 4: Comparison of parameter estimates based on single phylogeny, shared parameter estimates for 6 phylogenies together, and the original parameter values used for simulating trees via birth-death process.

We can also compare the AIC of the shared multi-clade model to the AIC of a set of single-tree models to check whether all the trees can be well explained by a single set of parameters. The AIC of a set of single-tree models can be obtained based on the fact that optimizing multiple functions separately yields the same results as optimizing a sum of them, each having different parameters. Based on this, such AIC can be calculated from the sum of log-likelihoods and model parameters of the single-tree models. The resulting AIC of the shared multi-clade model is lower than the AIC of the set of single-tree models, reflecting that the dataset was indeed simulated using one shared set of parameters.

```
aicmultibd=-2*(multiLHbd-2)
aicsinglebd=-2*(sum(singleLHbd)-2*6)
```

```
aicmultibd
## [1] 364.4781
aicsinglebd
## [1] 377.2007
```

Environment-dependent model

Here we generate a set of 6 phylogenetic trees using a birth death model with temperature dependent speciation rate. We use here the estimated diversification rates for *Primula*, which is the lineage for which we detected strongest dependence of diversification on past temperatures. The diversification process runs for 5.5 Ma and is controlled by the following parameters: baseline speciation rate lambda=0.1409249, exponential temperature dependence of speciation rate alpha=0.3602235 and extinction rate mu=9.859543e-08.

```
lambdaenv=0.1409249
alphaenv=0.3602235
muenv=9.859543e-08

data(InfTemp)

renvtreesim=function(lambdaenv,muenv,alphaenv){
  f.lamb = function(t, x, y) {y[1] * exp(x * y[2])}
  f.mu = function(t, x, y) {y[1]}
  sim_env_bd(InfTemp, f.lamb, f.mu, lamb_par = c(lambdaenv, alphaenv), mu_par =
muenv,
             time.stop = 5.5, return.all.extinct = F, prune.extinct = T)$tree
  }

tenvlist=mapply(renvtreesim,
rep(lambdaenv,6),rep(muenv,6),rep(alphaenv,6),SIMPLIFY = F)
```

We define a function *fit_envmulti* which is a shared-parameter equivalent of *fit_env* from *RPANDA* and serves as a wrapper of *fit_bdmulti* used in the previous example.

```
fit_envmulti=function (phylolist, env_data, tot_timelist, f.lamb, f.mu,
lamb_par,
          mu_par, df = NULL, flist, meth = "Nelder-Mead", cst.lamb = FALSE,
          cst.mu = FALSE, expo.lamb = FALSE, expo.mu = FALSE, fix.mu = FALSE,
          dt = 0, cond = "crown")
{
  if (is.null(df)) {
    df <- smooth.spline(x = env_data[, 1], env_data[, 2])$df
  }
  spline_result <- sm.spline(env_data[, 1], env_data[, 2],
                             df = df)
  env_func <- function(t) {
    predict(spline_result, t)
  }
  lower_bound_control <- 0.1
  upper_bound_control <- 0.1
  lower_bound <- min(env_data[, 1])
  upper_bound <- max(env_data[, 1])
  time_tabulated <- seq(from = lower_bound * (1 - lower_bound_control),
                        to = upper_bound * (1 + upper_bound_control), length.out
= 1 +
                          1e+06)
  env_tabulated <- env_func(time_tabulated)
  env_func_tab <- function(t) {
    b <- upper_bound * (1 + upper_bound_control)
    a <- lower_bound * (1 - lower_bound_control)
    n <- length(env_tabulated) - 1
    index <- 1 + as.integer((t - a) * n/(b - a))
    return(env_tabulated[index])
  }
  f.lamb.env <- function(t, y) {
    f.lamb(t, env_func_tab(t), y)
  }
  f.mu.env <- function(t, y) {
    f.mu(t, env_func_tab(t), y)
  }
  ####################################
  #here we use fit_bdmulti instead of fit_bd
  res <- fit_bdmulti(phylolist, tot_timelist, f.lamb.env, f.mu.env, lamb_par,
              mu_par, flist, meth=meth, cst.lamb, cst.mu, expo.lamb, expo.mu,
              fix.mu, dt, cond)
  ####################################
  res$model <- "environmental birth death"
  res$f.lamb <- function(t) {
    f.lamb(t, env_func_tab(t), res$lamb_par)
  }
  if (fix.mu == FALSE) {
    res$f.mu <- function(t) {
      f.mu(t, env_func_tab(t), res$mu_par)
    }
  }
  class(res) <- "fit.env"
  return(res)
}
```

Now we fit the environment-dependent model with shared parameters on the data.

```
#get total time for each tree
tot_timeenvfn=function(phylo){max(node.age(phylo)$ages)}
tot_timeenvlist=lapply(tenvlist,tot_timeenvfn)

#list of sampling proportion
fenvlist=as.list(rep(1,6))

#starting points for ML search
lamb_parenv<-c(runif(1,0,1),runif(1,0,1))
mu_parenv<-c(runif(1,0,lamb_parenv[1]/2))

#functions of time dependence (exponential and constant)
f.lambenv <-function(t,x,y){y[1] * exp(y[2] * x)}
f.muenv <-function(t,x,y){y[1]}

#fit
multienv=fit_envmulti(tenvlist, InfTemp, tot_timeenvlist,
                   f.lamb=f.lambenv,f.mu=f.muenv,
                   lamb_par=lamb_parenv,mu_par=mu_parenv,
                   cst.lamb = F,cst.mu = T,
                   flist=fenvlist, dt=1e-3)

multilambenv=abs(multienv$lamb_par[1])
multialphaenv=multienv$lamb_par[2]
multimuenv=abs(multienv$mu_par[1])
multiLHenv=multienv$LH
```

For comparison, we then fit the diversification model on each tree separately.

```
# fit
ferenv=function(phylo, tot_time){
  fit_env(phylo,InfTemp, tot_time,
          f.lamb=f.lambenv,f.mu=f.muenv,
          lamb_par=lamb_parenv,mu_par=mu_parenv,
          cst.lamb = F,cst.mu = T,
          f=1, dt=1e-3)
}
singleenv=mapply(ferenv,tenvlist,tot_timeenvlist)

#vectorize model characteristics from a list
singlelambenv=rep(0,6)
singlealphaenv=rep(0,6)
singlemuenv=rep(0,6)
singleLHenv=rep(0,6)

for(i in 1:6){
  tempres=singleenv[,i]
  singlelambenv[i]=abs(tempres$lamb_par[1])
  singlealphaenv[i]=tempres$lamb_par[2]
  singlemuenv[i]=abs(tempres$mu_par[1])
  singleLHenv[i]=tempres$LH
}
```

The plot of the parameter estimates suggests that the shared estimates of speciation, extinction rate and temperature-dependence of speciation closely match the original values, with single tree estimates being scattered around with larger variance. The aggregation of estimates around the original values suggests that also the environment-dependent diversification models are identifiable from the generated data.

```
plot(c(singlelambenv,singlealphaenv,singlemuenv)~c(rep(1,6),rep(2,6),rep(3,6)),
     xlim=c(0.5,3.5),ylim=c(0,1.2),xlab="",ylab="parameter estimate", pch=20,
xaxt="n")
axis(1, at = c(1,2,3), labels = c("lambda", "alpha", "mu"))
points(1,multilambenv, col="red", pch=19)
points(2,multialphaenv, col="red", pch=19)
points(3,multimuenv, col="red", pch=19)
points(1,lambdaenv, pch=4,cex=2)
points(2,alphaenv, pch=4,cex=2)
points(3,muenv, pch=4,cex=2)
legend(x='topright', legend=c("single-tree estimates", "shared estimate", "real
value"),
       col=c("black", "red", "black"), pch=c(20,19,4))
```

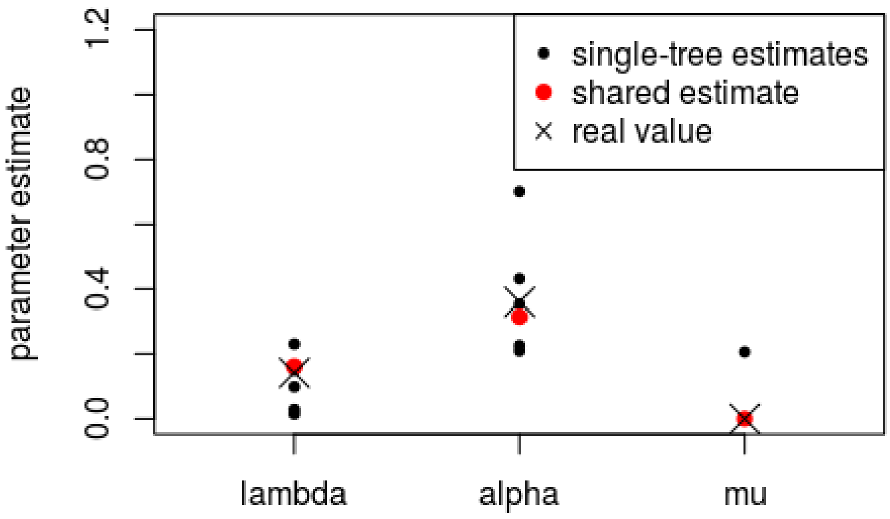

Supplementary Figure 5: Comparison of parameter estimates based on single phylogeny, shared parameter estimates for 6 phylogenies together, and the original parameter values used for simulating trees via environment-dependent birth-death process.

We then compare the AIC of the shared multi-clade model with the AIC of the set of single-tree models to check whether all the trees can be well explained by a single set of parameters. As it is shown below, the AIC of the shared multi-clade model is lower than the AIC of the set of single-tree models, reflecting that the environment-dependent set of trees was indeed generated using one shared set of parameters.

```
aicmultienv=-2*(multiLHbd-3)
aicsingleenv=-2*(sum(singleLHbd)-3*6)

aicmultienv
```

```
## [1] 366.4781
aicsingleenv
## [1] 389.2007
```

3 Sensitivity of temperature-dependent diversification models

In this appendix we perform a sensitivity analyses of temperature-dependent speciation models used in the main text, i.e. we explore type II errors of these models using simulated phylogenies with different strength of temperature dependence of speciation rate. Mirroring the analytical structure of the main text, we first analyze the sensitivity of single-lineage models and then of a multi-clade model operating on sets of 6 lineages.

Single-lineage models

To test the sensitivity of single lineage models, we simulated 700 phylogenies resembling the observed dataset, but with varying degree of temperature dependence of speciation rate. The speciation rate was dependent on temperature according to the formula lambda$*e^{alpha*t}$, where t is past reconstructed global temperature in °C[21]; and extinction was controlled by temperature-constant rate parameter mu. The phylogenies were simulated using randomly generated values of baseline speciation rate lambda and extinction rate mu corresponding to the maximum likelihood estimates from observed data, that is lambda between 0 and 1.35 and mu between 0 and value of respective lambda. Moreover, the simulations were conditioned to generate trees with crown age between 40 Ma BP and 3.2 Ma BP and having between 23 and 86 extant species, again reflecting the characteristics of the observed dataset. Such phylogenies were simulated 100 times for each value of temperature dependence of speciation alpha of 0, 0.1, 0.2, 0.3, 0.4, 0.5 and 0.6. alpha=0 represents independence of speciation on temperature, whereas alpha=0.6 represents a drop of Quaternary (2.6 Ma BP to present, mean global temperature 2 °C) speciation rate to approximately 24% of pre-Quaternary (5.2-2.6 Ma BP, mean global temperature 4.4 °C) level. The simulations were performed using *sim_env_bd* function from package RPANDA[19].

We fitted each of the simulated phylogenetic trees with constant birth death model and a temperature-dependent speciation model, and compared the AIC of the fits (AICdiff=AICbirthdeath-AICtempdep). In line with the main text, we evaluated sensitivity to two different types of result: “substantially supported” were the results where AICdiff>2, which corresponds to temperature-dependent model outperforming birth-death model even after addition of one completely non-informative parameter to the temperature-dependent model[22]; “marginally supported” were the results where AICdiff>0, which corresponds to at least slightly better informational performance of temperature-dependent model.

Our results (Fig. SM3.1) indicate that the single lineage models are sensitive to values of alpha=0.4 and higher, where the model properly identifies 69% of simulated phylogenies when referring to substantially supported result, and 82% of simulated phylogenies when referring to marginally supported result. Also for alpha=0.3, the phylogenies were properly categorized at least as marginally supported in 56% of cases. alpha=0.4 approximately corresponds to drop of Quaternary speciation rate to 38% of pre-Quaternary level, and alpha=0.3 to drop of Quaternary speciation rate to 50% of pre-Quaternary level.

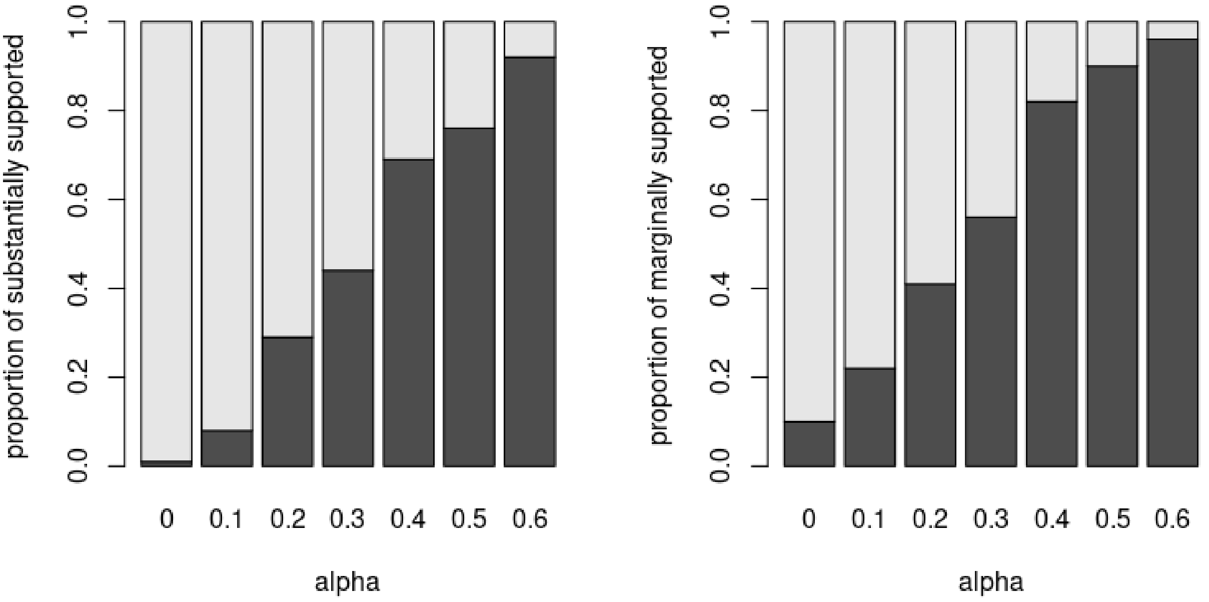

Supplementary Figure 6: Sensitivity of single-lineage temperature-dependent models. The left panel shows proportion of phylogenies identified as substantially supported as temperature-dependent (AICdiff>2), and the right panel shows the proportion of phylogenies identified as marginally temperature-dependent (AICdiff>0), for different values of temperature-dependence parameter alpha.

Multi-clade model

To explore sensitivity of multi-clade temperature-dependent diversification model, we simulated sets of 6 phylogenies, each of these sextuplets for 700 times. Similarly as for single-lineage models, phylogenies were simulated with exponentially temperature-dependent speciation, with sextuplet-specific randomly generated lambda between 0 and 1.35, mu between 0 and lambda; and were conditioned to crown age between 40 Ma BP and 3.2 Ma BP and 23 to 86 extant species. The sextuplets were simulated 100 times for each value of temperature dependence of speciation alpha of 0, 0.1, 0.2, 0.3, 0.4, 0.5 and 0.6. Substantial and marginal support for temperature-dependence was evaluated in the same way as as in the single-lineage models, using the multi-clade model described in the main text and Supplementary Methods 2.

The multi-clade model (Fig. SM3.2) was sensitive to values of alpha higher than 0.2, 67% of simulations with alpha=0.2 showed substantial support for temperature-dependent model, and 74% showed marginal support for temperature-dependent model. For alpha=0.3, the sensitivity was above 90% in both cases. alpha=0.2 approximately corresponds to drop of Quaternary speciation rate to 63% of pre-Quaternary level.

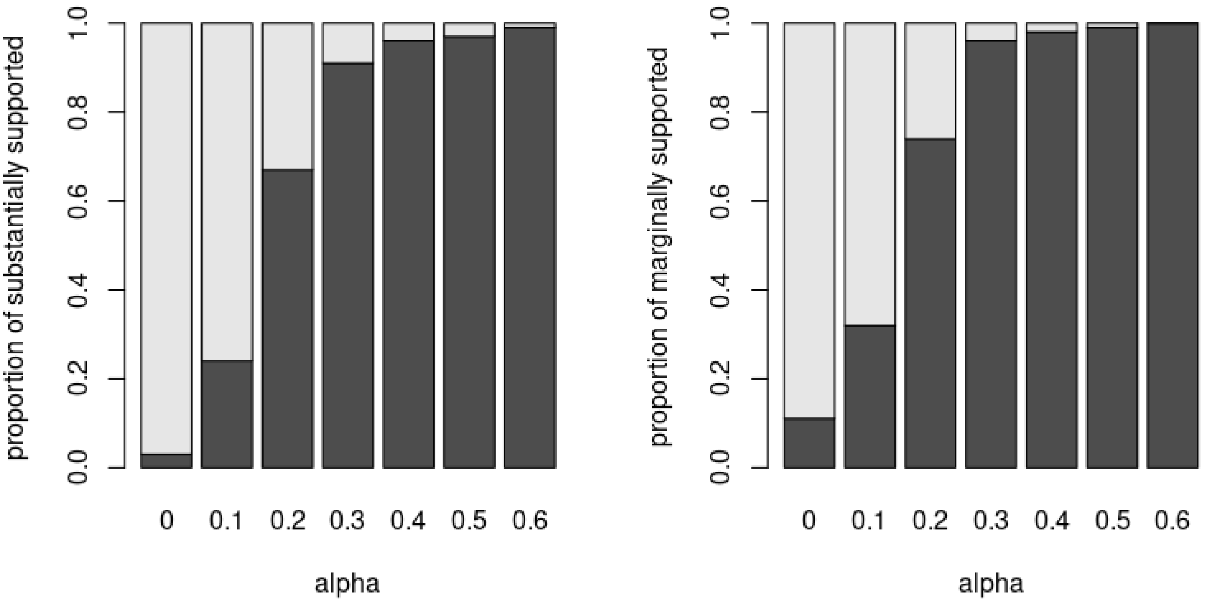

Supplementary Figure 7: Sensitivity of multi-clade temperature-dependent models. The left panel shows proportion of phylogenies substantially supported as temperature-dependent (AICdiff>2), and the right panel shows the proportion of phylogenies marginally temperature-dependent (AICdiff>0), for different values of temperature-dependence parameter alpha.

4 Multi-clade state-dependent diversification model R vignette

Introduction

This vignette demonstrates functioning of multi-clade state-dependent speciation-extinction (SSE) model used throughout the paper. The main idea behind this method is that the analyzed phylogenies with attributed tip states are *n* observations of the shared diversification and state evolution process and a likelihood of observing them together is thus a product of individual likelihood functions (Supplementary Equation 2).

$$\mathrm{L}_{\mathrm{alltogether}}(x) = \prod_{i=1}^{n} \mathrm{L}_i(x)$$

(Supplementary Equation 2)

This product likelihood function can be maximized to estimate the set of optimal diversification parameters shared by all the phylogenies. This approach was used previously[23,24] and is implemented via the procedure *combine* in the *diversitree* R package[25]. However, the proper functioning and sensitivity of this approach was not demonstrated in previous works. Here, we use phylogenies simulated from SSE processes with parameters detected in our dataset for evolutionary assembly across elevation belts and bedrocks to show that the parameter estimates of both single lineage and shared diversification models match the parameters of the generating process. We also demonstrate here that AICs are useful for assessing whether the shared model fits better the data than the single lineage models.

Elevation model

We first generate a set of 6 phylogenetic trees using a ClaSSE model with diversification and migration rates reflecting maximum likelihood estimates detected across elevational belts (lambda111=0.2972257, lambda222=0.3919789, mu=0.4725019, q13=0.2191140 and q23=1.1092566) containing 30 species.

```
library(diversitree)
## Loading required package: ape
library(gplots)
##
## Attaching package: 'gplots'
## The following object is masked from 'package:stats':
##
##     lowess
lambda111el=0.2972257 #speciation in mid elevation
lambda222el=0.3919789 #speciation in high elevation
muel=0.4725019 #extinction in both elevations
q13el=0.2191140 #migration from mid to high
q23el=1.1092566 #migration from high to mid

reltreesim=function(lambda111el,lambda222el,muel, q13el, q23el){
  repeat{
    t=tree.classe(c(lambda111el, 0, 0, 0, 0, 0, 0, 0, 0,
                lambda222el, 0, 0, 0, 0,
                lambda111el, 0, lambda222el, 0,
                muel, muel, 0, 0,
                q13el, 0, q23el,
                muel, muel),
              max.taxa=30, max.t=Inf, include.extinct=F,x0=NA)
    if (inherits(t, "phylo"))
                break
  }
  t
  }
tellist=mapply(reltreesim,
               rep(lambda111el,6),
               rep(lambda222el,6),
               rep(muel,6),
               rep(q13el,6),
               rep(q23el,6),
               SIMPLIFY = F)
```

Then we define a function turning ClaSSE into multivariate generalization of GeoSSE[23].

```
##############################################################
#function for restricting ClaSSE parameter space to be geographically
meaningful
formula_builder=function(set){
  #######################
  #a small function for sticking set elements into formula system
  #######################
  if (length(set)>1) {paste(set[2:length(set)], set[1],sep="~")}
}


zero_builder=function(set){
  #######################
  #a small function for sticking set elements into 0
  #######################
  if (length(set)>=1) {paste(set,"0",sep="~")}
}


restrict_classe2geosse=function(table, forbidden=NULL,
                                single.sympatry=F, no.sympatry=F,
                                single.vicariance=F, no.vicariance=F,
                                pooled.founder=F, single.founder=F,no.founder=F,
                                single.extinction=F, no.extinction=F,
                                pooled.migration=F, single.migration=F,
no.migration=F){

  #######################
  #input - states table
  #output - list of formulas to be used as constrain(lik,formulae=formulas)
  #######################

  #libraries
  library(diversitree)
  library(rje)

  #table into set representation
  tableset=list()
  for (i in 1:length(table[,1])) {tableset[[i]]=names(table)
[as.logical(table[i,])]}
  #get parameter names
  phy <- rcoal(100)
  names=names(starting.point.classe(phy, k=length(table[,1])))
  names=setdiff(names,forbidden)

  #parameter states from strings
  #############################
  #branching for k>10 DODELAT k>100
  if (length(table[,1])>10) {zp=1} else {zp=0}

  #lambdas
  lambdas=names[grep("lambda",names)]

  fromlambdas=as.numeric(substr(lambdas, 7, 7+zp*1))
  tolambdas1=as.numeric(substr(lambdas, 8+zp*1, 8+zp*2))
  tolambdas2=as.numeric(substr(lambdas, 9+zp*2, 9+zp*3))

  #mus
```

```
  mus=names[grep("mu",names)]
  frommus=as.numeric(substr(mus, 3, 3+zp*1))

  #qs
  qs=names[grep("q",names)]
  fromqs=as.numeric(substr(qs, 2, 2+zp*1))
  toqs=as.numeric(substr(qs, 3+zp*1, 3+zp*2))

  #biogeographic processes definition
  ###########################
  #sympatry
  sympatry_logical_list=list()
  sympatry_list=list()

  for (j in 1:length(names(table))){

    sympatry_logical_list[[j]]=rep(F,length(lambdas))

    for (i in 1:length(lambdas)){
      sympatry_logical_list[[j]]
[i]=(setequal(tableset[[fromlambdas[i]]],tableset[[tolambdas1[i]]]) &&
is.subset(tableset[[tolambdas2[i]]],tableset[[fromlambdas[i]]]) &&
setequal(tableset[[tolambdas2[i]]], names(table)[j])) | #2 is the offspring
species
        (setequal(tableset[[fromlambdas[i]]],tableset[[tolambdas2[i]]]) &&
is.subset(tableset[[tolambdas1[i]]],tableset[[fromlambdas[i]]]) &&
setequal(tableset[[tolambdas1[i]]], names(table)[j]))   #1 is the offspring
species
    }

    sympatry_list[[j]]=lambdas[sympatry_logical_list[[j]]]

  }

  #vicariance
  vicariance_logical=rep(F,length(lambdas))
  for (i in 1:length(lambdas)){

vicariance_logical[i]=(length(intersect(tableset[[tolambdas1[i]]],tableset[[tola
mbdas2[i]]]))==0 && #it is allopatry

setequal(tableset[[fromlambdas[i]]],union(tableset[[tolambdas1[i]]],tableset[[to
lambdas2[i]]]))) #union of offsprings is equal to ancestor
  }

  vicariance=lambdas[vicariance_logical]

  #founder (sensu bgb)
  founder_logical_list=list()
  founder_list=list()

  for (j in 1:length(names(table))){

    founder_logical_list[[j]]=rep(F,length(lambdas))

    for (i in 1:length(lambdas)){
      founder_logical_list[[j]]
[i]=(length(intersect(tableset[[tolambdas1[i]]],tableset[[tolambdas2[i]]]))==0
&& #it is allopatry
```

```
((setequal(tableset[[fromlambdas[i]]],tableset[[tolambdas1[i]]]) &&
setequal(tableset[[tolambdas2[i]]], names(table)[j])) | #2 is offspring species

(setequal(tableset[[fromlambdas[i]]],tableset[[tolambdas2[i]]]) &&
setequal(tableset[[tolambdas1[i]]], names(table)[j])))) #1 is offspring species
    }

    founder_list[[j]]=lambdas[founder_logical_list[[j]]]

  }

  #extinction
  extinction_q_logical_list=list()
  extinction_mu_logical_list=list()
  extinction_list=list()

  for (j in 1:length(names(table))){

    #local extinction
    extinction_q_logical_list[[j]]=rep(F,length(qs))

    for (i in 1:length(qs)){
      extinction_q_logical_list[[j]]
[i]=is.subset(tableset[[toqs[i]]],tableset[[fromqs[i]]]) && #offspring is
subset of ancestor
        setequal(setdiff(tableset[[fromqs[i]]],tableset[[toqs[i]]]),names(table)
[j]) #their difference is focal area
    }

    #global extinction
    extinction_mu_logical_list[[j]]=rep(F,length(mus))

    for (i in 1:length(mus)){
      extinction_mu_logical_list[[j]]
[i]=setequal(tableset[[frommus[i]]],names(table)[j]) #it is extinction in focal
area
    }


extinction_list[[j]]=c(qs[extinction_q_logical_list[[j]]],mus[extinction_mu_logi
cal_list[[j]]])

  }



  #migration
  migration_logical_list=list()
  migration_list=list()

  for (j in 1:length(names(table))){

    migration_logical_list[[j]]=rep(F,length(qs))

    for (i in 1:length(qs)){
      migration_logical_list[[j]]
[i]=is.subset(tableset[[fromqs[i]]],tableset[[toqs[i]]]) && #ancestor is a
subset of offspring
        setequal(setdiff(tableset[[toqs[i]]],tableset[[fromqs[i]]]),
names(table)[j]) #their difference is focal area
```

```
    }

    migration_list[[j]]=qs[migration_logical_list[[j]]]

  }

  #everything else and the forbidden parameters is a zero combination
  zerocombinations=c(setdiff(names, c(unlist(sympatry_list), vicariance,
unlist(founder_list), unlist(extinction_list),
unlist(migration_list))),forbidden)


  #build formulas
  ##############################

  #sympatry
  if (no.sympatry) {
    sympatry_formulas=zero_builder(unlist(sympatry_list))
  } else if (single.sympatry) {
    sympatry_formulas=formula_builder(unlist(sympatry_list))
  } else {
    sympatry_formulas=unlist(lapply(sympatry_list, formula_builder))
  }

  #vicariance
  if (no.vicariance) {
    vicariance_formulas=zero_builder(vicariance)
  } else if (single.vicariance) {
    vicariance_formulas=formula_builder(vicariance)
  } else {
    vicariance_formulas=NULL
  }

  #founder
  if (no.founder) {
    founder_formulas=zero_builder(unlist(founder_list))
  } else if (single.founder) {
    founder_formulas=formula_builder(unlist(founder_list))
  } else if (pooled.founder){
    founder_formulas=unlist(lapply(founder_list, formula_builder))
  } else {
    founder_formulas=NULL
  }

  #extinction
  if (no.extinction) {
    extinction_formulas=zero_builder(unlist(extinction_list))
  } else if (single.extinction) {
    extinction_formulas=formula_builder(unlist(extinction_list))
  } else {
    extinction_formulas=unlist(lapply(extinction_list, formula_builder))
  }

  #migration
  if (no.migration) {
    migration_formulas=zero_builder(unlist(migration_list))
  } else if (single.migration) {
    migration_formulas=formula_builder(unlist(migration_list))
  } else if (pooled.migration) {
    migration_formulas=unlist(lapply(migration_list, formula_builder))
```

```
  } else {
    migration_formulas=NULL
  }

  #not meaningful parameters
  zerocombinations_formulas=zero_builder(zerocombinations)

  #stick them in one list
  ##############################
  formulas=as.list(c( sympatry_formulas,
                      vicariance_formulas,
                      founder_formulas,
                      extinction_formulas,
                      migration_formulas,
                      zerocombinations_formulas))
  return(formulas)
}
```

In the next step, we fit the model with shared parameters on the generated data.

```
#generate ClaSSE likelihoods of each tree
mcel=function(tree){
  make.classe(tree, tree$tip.state, k=3,strict = F)
}
fellist=lapply(tellist,mcel)

#create joint likelihood of multi-clade model
likel=combine(fellist)

#restrict ClaSSE parametrization to GeoSSE-like
table=data.frame(mid=c(1,0,1),high=c(0,1,1))
formulasel=restrict_classe2geosse(table,
                                   no.vicariance = T,

                                   single.extinction = T,
                                   no.founder = T)
likmultiel=constrain(likel, formulae=formulasel)

#fit the model
p=starting.point.classe(tellist[[1]], k=3)

fitmultiel=find.mle(likmultiel, p[argnames(likmultiel)])
```

For comparison, we fit the diversification model on each tree separately.

```
#constrain likelihood function for each tree separately
scel=function(lik){
  constrain(lik, formulae=formulasel)
  }
slikellist=lapply(fellist,scel)

#fit each tree
fiel=function(lik){
  find.mle(lik, p[argnames(likmultiel)])
  }
sfitellist=lapply(slikellist,fiel)
```

The plot of the shared and single tree estimates shows that the shared estimates closely match the original value and that the single tree estimates are scattered around with larger variance. The aggregation of estimates around the original values suggests that the shared multi-clade model is correctly identifiable from the generated data. The single tree models are theoretically identifiable as well, but the large dispersion around the original values suggests that estimates of individual phylogenies may fairly deviate from the generating values for the considered size of phylogeny (30 species).

```
plot(NULL, xlim=c(0,6),ylim=c(0,2),xaxt="n", ylab="parameter estimate",xlab="")
for (i in 1:6){
  points(1:5,sfitellist[[i]]$par, pch=20)
}
points(1:5, fitmultiel$par, col="red", pch=19)
points(1:5, c(lambda111el,lambda222el, q13el, q23el,muel), pch=4,cex=2)
angleAxis(1,srt=40,at = 1:5,
          labels = c("speciation in mid", "speciation in high", "migration to
high", "migration to mid", "extinction"),
          offset=0.1)
legend(x='topleft',
       legend=c("single-tree estimates", "shared estimate", "real value"),
       col=c("black", "red", "black"), pch=c(20,19,4))
```

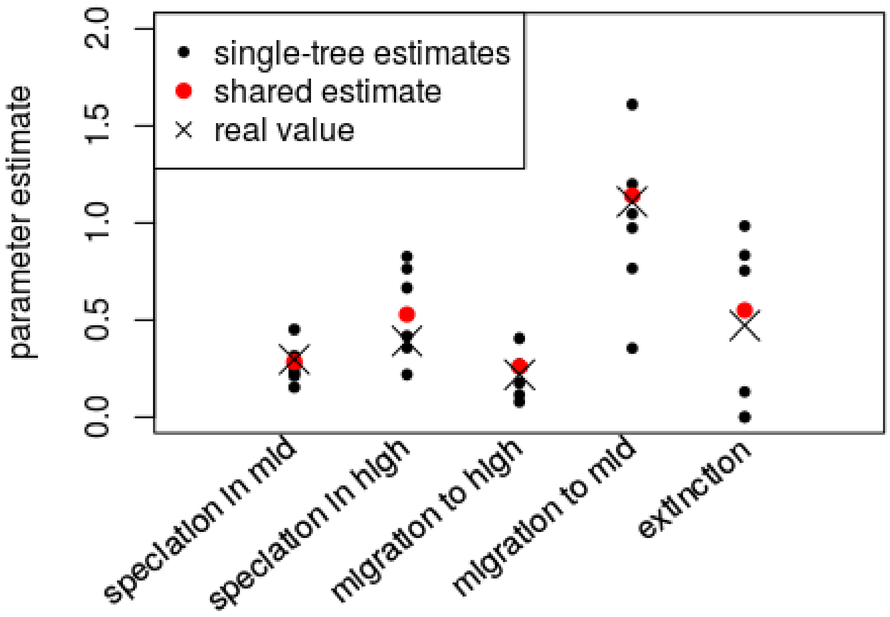

Supplementary figure 8: Comparison of parameter estimates based on single phylogeny, shared parameter estimates for 6 phylogenies together, and the original parameter values used for simulating trees via state-dependent diversification process without state-change speciation.

Then, we compare the AIC of the shared multi-clade model to the AIC of single-tree models to assess their relative goodness of fit. The AIC of single-tree models can be obtained based on the fact that optimizing multiple functions separately yields the same results as optimizing a sum of them, each having different parameters. Based on this, the AICs can be calculated from the sum of log-likelihoods and model parameter counts of the single-tree models. The resulting AIC of the shared multi-clade model is lower than the AIC of the set of single-tree models, reflecting that the dataset was indeed generated using one shared set of parameters.

```
aicmultiel=-2*(fitmultiel$lnLik-5)

slhel=rep(0,6)
for (i in 1:6){slhel[i]=sfitellist[[i]]$lnLik}
aicsingleel=-2*(sum(slhel)-5*6)

aicmultiel
## [1] 1046.624
aicsingleel
## [1] 1064.749
```

Bedrock model

In the second part, we generate a set of 6 phylogenetic trees using a ClaSSE model with diversification and migration rates reflecting maximum likelihood estimates detected across bedrock belts (lambda111=0.2051032, lambda222=0.4424083, lambda312=0.4527579, mu=0.4496823, q13=0.1406433 and q23=0.5780840) containing 30 species. It should be noted that, unlike for elevation, the bedrock model contains a state-change speciation term (lambda312).

```
lambda111be=0.2051032 #speciation on calcareous bedrock
lambda222be=0.4424083 #speciation on siliceous bedrock
lambda312be=0.4527579 #speciation with split to calcareous and species
mube=0.4496823 #extinction on both bedrocks
q13be=0.1406433 #migration from calcareous to siliceous
q23be=0.5780840 #migration from siliceous to calcareous

rbetreesim=function(lambda111be,lambda222be, lambda312be,mube, q13be, q23be){
  repeat{
    t=tree.classe(c(lambda111be, 0, 0, 0, 0, 0, 0, 0, 0,
                lambda222be, 0, 0, 0,
                lambda312be,
                lambda111be, 0, lambda222be, 0,
                mube, mube, 0, 0,
                q13be, 0, q23be,
                mube, mube),
              max.taxa=30, max.t=Inf, include.extinct=F,x0=NA)
    if (inherits(t, "phylo"))
                break
  }
  t
  }
tbelist=mapply(rbetreesim,
               rep(lambda111be,6),
               rep(lambda222be,6),
               rep(lambda312be,6),
               rep(mube,6),
               rep(q13be,6),
               rep(q23be,6),
               SIMPLIFY = F)
```

To fit the model with shared parameters, we use the same procedure as for elevational belts, that is, we use the function *combine* from *diversitree* to multiply the likelihood function.

```
#generate ClaSSE likelihoods of each tree
mcbe=function(tree){
  make.classe(tree, tree$tip.state, k=3,strict = F)
}
fbelist=lapply(tbelist,mcbe)

#create joint likelihood of multi-clade model
likbe=combine(fbelist)

#restrict ClaSSE parametrization to GeoSSE-like
table=data.frame(calc=c(1,0,1),cilic=c(0,1,1))
formulasbe=restrict_classe2geosse(table,
                                   single.extinction = T,
                                   no.founder = T)
likmultibe=constrain(likbe, formulae=formulasbe)

#fit the model
p=starting.point.classe(tbelist[[1]], k=3)

fitmultibe=find.mle(likmultibe, p[argnames(likmultibe)])
```

For comparison, we fit the model on each tree separately.

```
#constrain likelihood function for each tree separately
scbe=function(lik){
  constrain(lik, formulae=formulasbe)
  }
slikbelist=lapply(fbelist,scbe)

#fit each tree
fibe=function(lik){
  find.mle(lik, p[argnames(likmultibe)])
  }
sfitbelist=lapply(slikbelist,fibe)
```

The plot of the shared and single tree estimates shows that the shared estimates closely match the original value and the single tree estimates are scattered around with large variance, similarly as for the elevation SSE model. The shared multi-clade model of evolutionary assembly across bedrocks is thus correctly identifiable from the generated data, including the state-change speciation term. The same is true for single tree models, but in phylogenies of size ~30 species, there are considerable deviations of individual tree estimates from the generating values.

```
plot(NULL, xlim=c(0,7),ylim=c(0,2),xaxt="n", ylab="parameter estimate",xlab="")
for (i in 1:6){
  points(1:6,sfitbelist[[i]]$par, pch=20)
}
points(1:6, fitmultibe$par, col="red", pch=19)
points(1:6, c(lambda111be,lambda222be,lambda312be, q13be, q23be,mube),
pch=4,cex=2)

angleAxis(1,srt=40,at = 1:6,
          labels = c("speciation in cal", "speciation in sil","speciation
split", "migration to sil", "migration to cal", "extinction"),
          offset=0.1)
legend(x='topleft',
       legend=c("single-tree estimates", "shared estimate", "real value"),
       col=c("black", "red", "black"), pch=c(20,19,4))
```

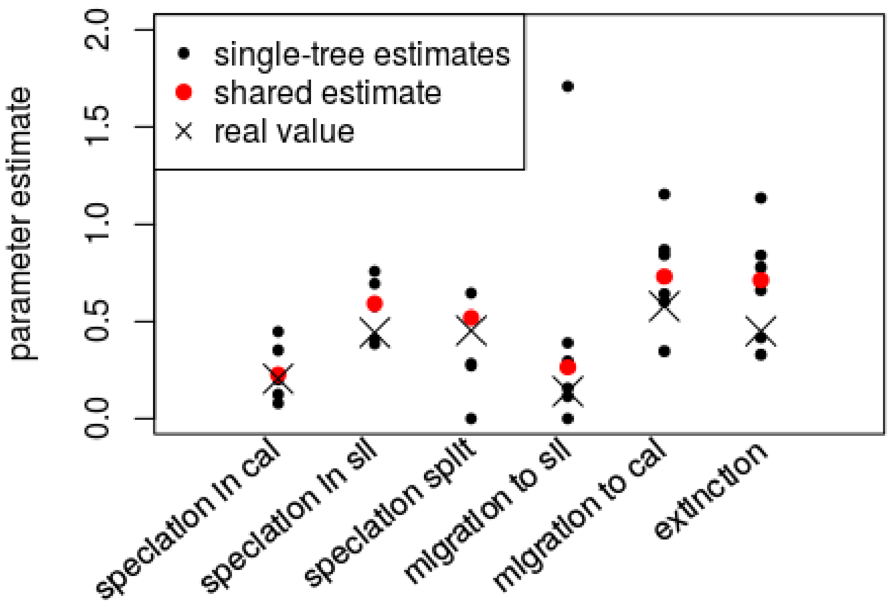

Supplementary figure 9: Comparison of parameter estimates based on single phylogeny, shared parameter estimates for 6 phylogenies together, and the original parameter values used for simulating trees via state-dependent diversification process with state-change speciation.

Also here, we can also compare the AIC of the shared multi-clade model to the AIC of single-tree models to verify whether the model with a shared set of parameters fits better the data. The resulting AIC of the shared multi-clade model is lower than the AIC of the set of single-tree models, reflecting that the dataset was indeed generated using one shared set of parameters.

```
aicmultiel=-2*(fitmultiel$lnLik-5)

slhel=rep(0,6)
for (i in 1:6){slhel[i]=sfitellist[[i]]$lnLik}
aicsingleel=-2*(sum(slhel)-5*6)

aicmultiel
## [1] 1046.624
aicsingleel
## [1] 1064.749
```

5 Compilation of geographic information

As the small scale geographic regions, we used operative geographic units in Flora Alpina[26] for the Alps, and mountain regions based on Körner et al. 2017[27] for other European mountains, with subsequent modifications that better reflect structuring of biogeographic information in local floristic literature.
Specifically, we merged:

- Pennines and Cambrian Mountains to England
- MacGillycuddy's Reeks and Wicklow Mountains to Ireland
- Vosges, Black Forest and Jura Mountains to Rhine Valley
- Grampian Mountains, Northwestern Highlands and Southern Uplands to Scotland

We joined:

- Basque Mountains and Tras-os-montes to Cantabrian Mountains
- Korab, Sar Planina and Jablanica to Dinaric Alps
- Kontovounia, Tayetos Oros, Crete and Parnon Oros to Peloponnisos
- Gribe, Mali i Gjere, Mount Nemercke, Mount Olympus and Mount Othris to Pindos
- Osogovo, Maleshevo, Belasica, Voras Mountains, Rila, Pirin and Pangaion Hills to Rhodope mountains

We renamed:

- Balkan Mountains to Stara Planina

We divided:

- Carpathian Mountains to Western Carpathians and South Eastern Carpathians along the border between Slovakia and Ukraine

We newly defined:

- lower mountain ranges in Czechia not included into Western Carpathians as Sudetes

In addition to this, whole Africa, Middle East (including Caucasus) and Arctic (including Siberian mountains) were represented by one region each, accommodating species with ranges extending out of Europe. Species occurring in lowlands out of mountain regions were always attributed to geographically closest mountain region.

The 5 major geographic regions were defined as sets of small scale geographic regions, specifically:

- Alps: Alps, Massif central and Rhine valley
- Apeninnes: Apennines, Corsica, Sardinia and Sicily
- Balkans: Dinaric Alps, Rhodopes, Stara planina, Peloponnisos, Pindos and Middle East (the latter region was merged within the Balkans because no focal endemic species and strong floristic connections)
- Carpathians: Carpathians, Sudetes, all northern European regions and Arctic (the latter region was merged within the Carpathians because very few focal endemic species and strong floristic connections)
- Iberian mountains: all mountains on Iberian Peninsula, Mallorca, Madeira and northern Africa (the latter two regions were merged within Iberian mountains because of very few focal endemic species and strong floristic connections)

For resulting table of regions for each species, see Supplementary Data 4.

6 Species sampling and taxonomic treatment

Our ingroup sampling included 38 samples of 26 species for *Androsace* sect. *Aretia* (which includes in total 29 species excluding subgenus *Douglasia*, see below), 80 samples of 45 species for *Campanula* sect. *Heterophylla* (in total 50 species), 31 samples of 28 species for *Gentiana* sections *Gentiana, Ciminalis and Calanthianae* (in total 35 species; all 3 sections considered together, see below), 33 samples of 27 species for *Phyteuma* (in total 27 species), 28 samples of 24 species for *Primula* sect. *Auriculata* (in total 24 species), 69 samples of 62 species for *Saxifraga* sect. *Saxifraga* (in total 86 species). Concerning outgroups, we included 49 outgroup samples of Campanulaceae, 14 outgroup samples of Gentianaceae, 95 outgroup samples of Primulaceae, and 49 and 6 outgroup samples of Saxifragaceae and Grossulariaceae, respectively. In total our dataset contains 492 samples. The large majority of dataset was collected in the field, but 35 samples come from herbarium specimens, and 22 samples come from individuals cultivated in botanical gardens from seeds collected in the field. Please see Supplementary Data 1 and 2 for details on sample identities and counts.

We detail below the taxonomic literature and molecular phylogenetic studies that we took in account to generate the list of ingroup species and subspecies with unambiguously attributable morphological description, ecology and geographic range. For all the lineages, we took in account information from regional floristic literature: Flora Alpina[26], Flora Iberica[28], Flóra Slovenska[29], Wildpflanzen Siebenbürgens[30] and Flora Srbije[31]. In addition to this, we took in account specific taxonomic literature for each of the lineages, specifically:

- For *Androsace* sect. *Aretia* we used Schneeweiss et al. 2004[32], Schonswetter et al. 2009[33], Boucher et al. 2012[34], Schonswetter et al. 2015[35] and Boucher et al. 2015[15].
-For *Campanula* sect. *Heterophylla* we used Mansion et al. 2012[1], Kovačić 2004[36] and Fenaroli et al. 2013[37]. As this lineage is the most complex one of those included in this study from the taxonomic point of view, we established a specific collaboration with Kristýna Šemberová (co-author of this study) because she is currently conducting a detailed taxonomic revision of the species of this group. For controverted species, we included several individuals to check for species monophyly. In addition, a specific study integrating molecular and morphological data of Pyrenean species has been conducted for the taxonomic revision and species delimitation of controverted species endemic to this area[38].
- For *Gentiana* we used Favre 2015[4], and specifically for section *Gentiana* we used Rossi 2011[39] and for section *Calathianae* we used Hämmerli 2007[40].
-For *Phyteuma we used* Schneeweiss et al. 2013[41].
- For *Primula* sect. *Auriculata* we used Zhang and Kadereit 2004[42] and Boucher et al. 2015[15].
- For *Saxifraga* sect. *Saxifraga* we used Vargas 2000[43], Webb and Gornall 1989[44] and Tkach et al. 2015[45].

Where available and specifically for controverted taxa, we used more than one sample per species for constructing phylogenies. To turn the sample topologies into species trees, we applied several specific treatments:

- We selected the tips of paraphyletic species to conserve most recent splitting node: 4 species in our phylogenies (*Gentiana lutea* ssp. *lutea*, *Androsace halleri* ssp. *halleri*, *Androsace cylindrica* ssp. *hirtella*, *Saxifraga glabella*) proved to be paraphyletic, i.e. with another species arising from within subtree of their samples. This phenomenon may be caused by incomplete lineage sorting, but may also reflect evolutionary reality of budding speciation within these lineages. In order to keep the most realistic splitting date between such hierarchically organized species, we selected the samples of paraphyletic species in order to conserve the most recent splitting node.
- We pruned the sample of *Campanula witasekiana* from Eastern Alps: *Campanula witasekiana* was a seemingly polyphyletic species in our dataset, with one sample from Dinaric mountains in Bosnia and another from Austrian Alps, lying in different parts of *Campanula* sect. *Heterophylla* phylogeny. Given the inconsistency between species descriptions by botanical communities in the Balkans and in Austria, and the fact that the type locality of species is in Bosnia, we assumed that the description *C. witasekiana* from Eastern Alps is erroneous and decided to keep the sample of *C. witasekiana* from Bosnia in our species tree.
- We pruned those samples determined in the field that could not be later verified without doubt from herbarium sheets. In other words, those samples identified in the field as belonging to the species complexes of *Campanula rotundifolia, C. scheuchzerii*, *Gentiana verna* or *Primula auricul*a that could not be without

doubts attributed to the taxon identified in the field were not considered when constructing the species trees. These *sensu lato* samples are marked with the text “sl” following scientific name in the accession tables.

In addition to this, we took two specific taxonomic treatments:
- We considered *Gentiana* sect. *Gentiana*, *Ciminalis* and *Calathianae* as a single lineage. Monophyly of these lineages was previously suggested[4]. We found that these three European sections of *Gentiana* form a monophyletic clade with a posterior probability of 1, so we treat them as a single lineage.
- We pruned the subgenus *Douglasia* from the *Androsace* sect. *Aretia* phylogeny: The subgenus *Douglasia* is monophyletic and endemic to north America. It thus represents, at most, a single speciation event in relation to the European mountain system, while the diversification of this subgenus took place in North America, which is out of the geographic scope of our study.

Supplementary Note – The PhyloAlps consortium

Sébastien Lavergne[1], Eric Coissac[1], Cristina Roquet[1,4], Martí Boleda[1], Wilfried Thuiller[1], Ludovic Gielly[1], Pierre Taberlet[1], Delphine Rioux[1], Frédéric Boyer[1], Anthony Hombiat[1], Bruno Bzeznick[1], Adriana Alberti[5,6], France Denoeud[5], Patrick Wincker[5], Christophe Perrier[7], Rolland Douzet[7], Maxime Rome[7], Jean-Gabriel Valay[7], Serge Aubert[7], Niklaus Zimmermann[9], Rafael O. Wüest[9], Sonia Latzin[9], John Spillmann[9], Linda Feichtinger[9], Jérémie Van Es[11], Luc Garraud[11], Jean-Charles Villaret[11], Sylvain Abdulhak[11], Véronique Bonnet[11], Stéphanie Huc[11], Noémie Fort[11], Thomas Legland[11], Thomas Sanz[11], Gilles Pache[11], Alexis Mikolajczak[11], Virgile Noble[12], Henri Michaud[12], Benoît Offerhaus[12], Cédric Dentant[13], Pierre Salomez[13], Richard Bonet[13], Thierry Delahaye[14], Marie-France Leccia[15], Monique Perfus[15], Stefan Eggenberg[16], Adrian Möhl[16], Bogdan-Iuliu Hurdu[17], Paul-Marian Szatmari[17], Mihai Pușcaș[18], Patrik Mráz[3], Michał Ronikier[19]

[11] Conservatoire Botanique National Alpin, Domaine de Charance, FR-05000 Gap, France
[12] Conservatoire Botanique National Méditerranéen, FR-83400 Hyères, France
[13] Parc National des Ecrins, FR-05000 Gap, France
[14] Parc National de la Vanoise, FR-73000 Chambery, France
[15] Parc National du Mercantour, FR-06006 Nice Cedex 1, France
[16] Info-Flora – Centre national de données et d'informations sur la flore de Suisse, Genève, CH-3001 Bern, Switzerland
[17] Institute of Biological Research, National Institute of Research and Development for Biological Sciences, RO-400015 Cluj-Napoca, Romania
[18] Department of Taxonomy and Ecology, Faculty of Biology and Geology and Al. Borza Botanic Garden - Babeș-Bolyai University, RO-400015 Cluj-Napoca, Romania
[19] W. Szafer Institute of Botany, Polish Academy of Sciences, PL-31512 Kraków, Poland

Supplementary References

1. Mansion, G. *et al.* How to handle speciose clades? Mass taxon-sampling as a strategy towards illuminating the natural history of Campanula (Campanuloideae). *PLoS One* **7**, (2012).
2. Łańcucka-Środoniowa, M. Macroscopic plant remains from the freshwater Miocene of the Nowy Sącz Basin (West Carpathians, Poland). *Acta Palaeobot.* **1**, 3–117 (1979).
3. Bell, C. D., Soltis, D. E. & Soltis, P. S. The age and diversification of the angiosperms re-revisited. *Am. J. Bot.* **97**, 1296–1303 (2010).
4. Favre, A. *et al.* Out-of-Tibet: the spatio-temporal evolution of Gentiana (Gentianaceae). *J. Biogeogr.* **43**, 1967–1978 (2016).
5. Pirie, M. D., Litsios, G., Bellstedt, D. U., Salamin, N. & Kissling, J. Back to Gondwanaland: Can ancient vicariance explain (some) Indian ocean disjunct plant distributions? *Biol. Lett.* **11**, (2015).
6. *The Gentianaceae - Volume 1: Characterization and Ecology*. (Springer, 2014).
7. Graham, A. Lisianthius pollen from the Eocene of Panama. *Ann. Missouri Bot. Gard.* **71**, 987–993 (1984).
8. Merckx, V. S. F. T. *et al.* Phylogenetic relationships of the mycoheterotrophic genus Voyria and the implications for the biogeographic history of Gentianaceae. *Am. J. Bot.* **100**, 712–721 (2013).
9. Xing, Y. & Ree, R. H. Uplift-driven diversification in the Hengduan Mountains, a temperate biodiversity hotspot. *Proc. Natl. Acad. Sci.* **114**, E3444–E3451 (2017).
10. Czaja, A. *Paläokarpologische Untersuchungen von Taphozönosen des Unter- und Mittelmiozäns aus dem Braunkohlentagebau Berzdorf/Oberlausitz (Sachsen)*. (Schweizerbart, 2003).
11. Friis, E. M., Pedersen, K. R. & Crane, P. R. Cretaceous diversification of angiosperms in the western part of the Iberian Peninsula. *Rev. Palaeobot. Palynol.* **162**, 341–361 (2010).
12. Dorofeev, P. I. & Takhtajan, A. L. Primulaceae. in *Oznovij Paleontologii* 517–518 (Izdavatelstvo Akademii nauk SSSR, 1963).
13. Dorofeev, P. I. *Treticnye flory zapadnoj Sibiri*. (Izdavatelstvo Akademii nauk SSSR, 1963).
14. Collinson, M. E. *Fossil plants of the London Clay*. (Willey-Blackwell, 1984).
15. Boucher, F. C., Zimmermann, N. E. & Conti, E. Allopatric speciation with little niche divergence is common among alpine Primulaceae. *J. Biogeogr.* **43**, 591–602 (2016).
16. Ebersbach, J. *et al.* In and out of the Qinghai-Tibet Plateau: divergence time estimation and historical biogeography of the large arctic-alpine genus Saxifraga L. *J. Biogeogr.* **44**, 900–910 (2017).
17. Hermsen, E. The fossil record of Iteaceae and Grossulariaceae in the cretaceous and tertiary of the United States and Canada. (Cornell University, 2005).
18. Hughes, N. F. *The Enigma of Angiosperm Origin*. (Cambridge Paleobiology Series, 1994).
19. Morlon, H. *et al.* RPANDA: An R package for macroevolutionary analyses on phylogenetic trees. *Methods Ecol. Evol.* **7**, 589–597 (2016).

20. Louca, S. & Pennell, M. W. Extant timetrees are consistent with a myriad of diversification histories. *Nature* **580**, 502–505 (2020).

21. Zachos, J. C., Dickens, G. R. & Zeebe, R. E. An early Cenozoic perspective on greenhouse warming and carbon-cycle dynamics. *Nature* **451**, 279–283 (2008).

22. Burnham, K. & Anderson, D. *Model Selection and Multimodel Inference*. (Springer, 2002).

23. Goldberg, E. E., Lancaster, L. T. & Ree, R. H. Phylogenetic inference of reciprocal effects between geographic range evolution and diversification. *Syst. Biol.* **60**, 451–465 (2011).

24. Anacker, B. L., Whittall, J. B., Goldberg, E. E. & Harrison, S. P. Origins and consequences of serpentine endemism in the California flora. *Evolution* **65**, 365–376 (2011).

25. Fitzjohn, R. G., Maddison, W. P. & Otto, S. P. Estimating trait-dependent speciation and extinction rates from incompletely resolved phylogenies. *Syst. Biol.* **58**, 595–611 (2009).

26. Aeschimann, D., Lauber, K., Moser, D. M. & Theurillat, J. P. *Flora Alpina*. (Editions Belin, 2004).

27. Körner, C. *et al.* A global inventory of mountains for bio-geographical applications. *Alp. Bot.* **127**, 1–15 (2017).

28. *Flora Iberica*. (Real Jardin Botanico CSIC, 2012).

29. *Flóra Slovenska*. (Vydavateľstvo Slovenskej akadémie vied, 2012).

30. Speta, E. & Rákosy, L. *Wildpflanzen Siebenbürgen*. (Naturhistorisches Museum Wien, 2010).

31. *Flora Srbije*. (Srpska akademija nauka i umetnosti, 1992).

32. Schneeweiss, G. M., Schönswetter, P., Kelso, S. & Niklfeld, H. Complex biogeographic patterns in Androsace (Primulaceae) and related genera: Evidence from phylogenetic analyses of nuclear internal transcribed spacer and plastid trnL-F sequences. *Syst. Biol.* **53**, 856–876 (2004).

33. Schönswetter, P. & Schneeweiss, G. M. Androsace komovensis sp. nov., a long mistaken local endemic from the southern Balkan Peninsula with biogeographic links to the Eastern Alps. *Taxon* **58**, 544–549 (2009).

34. Boucher, F. C. *et al.* Reconstructing the origins of high-alpine niches and cushion life form in the genus Androsace sl (Primulaceae). *Evolution* **66**, 1255–1268 (2012).

35. Schönswetter, P., Magauer, M. & Schneeweiss, G. M. Androsace halleri subsp. nuria Schönsw. & Schneew. (Primulaceae), a new taxon from the eastern Pyrenees (Spain, France). *Phytotaxa* **201**, 227–232 (2015).

36. Kovacic, S. The genus Campanula L. (Campanulaceae) in Croatia, circum-Adriatic and west Balkan region. *Acta Bot. Croat.* **63**, 171–202 (2004).

37. Fenaroli, F., Pistarino, A., Peruzzi, L. & Cellinese, N. Campanula martinii (Campanulaceae), a new species from northern Italy. *Phytotaxa* **111**, 27–38 (2013).

38. Roquet, C. *et al.* Evolutionary origins and species delineation of the two Pyrenean endemics Campanula jaubertiana and C. andorrana (Campanulaceae): evidence for transverse alpine speciation. *Alp. Bot.* (2021)

39. Rossi, M. Taxonomy, phylogeny and reproductive ecology of Gentiana lutea L. (University in Bologna, 2011).

40. Hämmerli, M. Molecular Aspects in Systematics of Gentiana Sect. Calathianae Froel. (Université de Neuchâtel, 2007).

41. Schneeweiss, G. M. *et al.* Molecular phylogenetic analyses identify Alpine differentiation and dysploid chromosome number changes as major forces for the evolution of the European endemic Phyteuma (Campanulaceae). *Mol. Phylogenet. Evol.* **69**, 634–652 (2013).

42. Zhang, L. B. & Kadereit, J. W. Classification of Primula sect. Auricula (Primulaceae) based on two molecular data sets (ITS, AFLPs), morphology and geographical distribution. *Bot. J. Linn. Soc.* **146**, 1–26 (2004).

43. Vargas, P. Plant Systematics and Evolution A phylogenetic study of Saxifraga sect. Saxifraga (Saxifragaceae) based on nrDNA ITS sequences. *Plant Syst. Evol* **223**, 59–70 (2000).

44. Webb, D. A. & Gornall, R. J. *Saxifrages of Europe*. (Timber Press, 1989).

45. Tkach, N. *et al.* Molecular phylogenetics, morphology and a revised classification of the complex genus Saxifraga (Saxifragaceae). *Taxon* **64**, 1159–1187 (2015).
