# Supplementary material for: Tempo and drivers of plant diversification in the European mountain system
Source: Nat Commun. 2022 May 18;13:2750. doi: 10.1038/s41467-022-30394-5 (PMC9117672; doi:10.1038/s41467-022-30394-5)
Supplement: Supplementary file 3 — Description of Additional Supplementary Files [file 41467_2022_30394_MOESM3_ESM.pdf]

Description of Additional Supplementary Files

Supplementary Data 1 Accession table. A table containing information about database identity, taxonomy and sampling information of each sample.

Supplementary Data 2 Sample to species conversion table. A table coding sample identities used for construction of species trees, completeness of sample alignments and linkage of sample identities to phylogenies.

Supplementary Data 3 Genomic regions. A table of coding and non-coding regions used for phylogenies of the four families.

Supplementary Data 4 Ecological and geographic information. A table of ecological (bedrock and elevation belt) and geographic (coarse and fine scale) information for each ingroup species.

Supplementary Software 1 Maximum credibility species trees. A list of maximum credibility species trees for each clade in Newick format.

Supplementary Software 2 Alternative maximum credibility species trees. A list of maximum credibility species trees in Newick format using alternative dating approaches and interpretation of fossil record.
