# Supplementary material for: Tempo and drivers of plant diversification in the European mountain system
Source: Nat Commun. 2022 May 18;13:2750. doi: 10.1038/s41467-022-30394-5 (PMC9117672; doi:10.1038/s41467-022-30394-5)
Supplement: Supplementary file 10 — Reporting Summary [file 41467_2022_30394_MOESM10_ESM.pdf]

nature portfolio

Corresponding author(s): Jan Smyčka

Last updated by author(s): 2022-21-04

Reporting Summary

Nature Portfolio wishes to improve the reproducibility of the work that we publish. This form provides structure for consistency and transparency in reporting. For further information on Nature Portfolio policies, see our Editorial Policies and the Editorial Policy Checklist.

Statistics

For all statistical analyses, confirm that the following items are present in the figure legend, table legend, main text, or Methods section.

n/a	Confirmed	
☐	☒	The exact sample size (n) for each experimental group/condition, given as a discrete number and unit of measurement
☐	☒	A statement on whether measurements were taken from distinct samples or whether the same sample was measured repeatedly
☒	☐	The statistical test(s) used AND whether they are one- or two-sided<br>*Only common tests should be described solely by name; describe more complex techniques in the Methods section.*
☐	☒	A description of all covariates tested
☐	☒	A description of any assumptions or corrections, such as tests of normality and adjustment for multiple comparisons
☐	☒	A full description of the statistical parameters including central tendency (e.g. means) or other basic estimates (e.g. regression coefficient) AND variation (e.g. standard deviation) or associated estimates of uncertainty (e.g. confidence intervals)
☒	☐	For null hypothesis testing, the test statistic (e.g. F, t, r) with confidence intervals, effect sizes, degrees of freedom and P value noted<br>*Give P values as exact values whenever suitable.*
☐	☒	For Bayesian analysis, information on the choice of priors and Markov chain Monte Carlo settings
☐	☒	For hierarchical and complex designs, identification of the appropriate level for tests and full reporting of outcomes
☒	☐	Estimates of effect sizes (e.g. Cohen's d, Pearson's r), indicating how they were calculated

Our web collection on statistics for biologists contains articles on many of the points above.

Software and code

Policy information about availability of computer code

Data collection	No automated data acquisition was performed in this study.
Data analysis	The analyses were performed with following open source software:<br>-Python 3<br>-Beast 2.6.2<br>-Org.Asm 1.0.3<br>-R 3.5.2<br>-RPANDA 1.5<br>-diversitree 0.9-11<br>-spaa 0.2.2<br>-fastX 0.0.13<br>-MACSE<br>-Gblocks 0.91<br>-FasConCat<br>-Mafft 7<br>-bModeltest 1.2.0<br>and custom R code reported in Methods and Supplementary methods, and deposited here doi:10.5281/zenodo.6341727.

For manuscripts utilizing custom algorithms or software that are central to the research but not yet described in published literature, software must be made available to editors and reviewers. We strongly encourage code deposition in a community repository (e.g. GitHub). See the Nature Portfolio guidelines for submitting code & software for further information.

Data

Policy information about availability of data

All manuscripts must include a data availability statement. This statement should provide the following information, where applicable:

- Accession codes, unique identifiers, or web links for publicly available datasets
- A description of any restrictions on data availability
- For clinical datasets or third party data, please ensure that the statement adheres to our policy

Raw genomic data generated in this study are available in European Nucleotide Archive under study accession codes https://www.ebi.ac.uk/ena/browser/view/PRJEB43865, https://www.ebi.ac.uk/ena/browser/view/PRJEB48693, https://www.ebi.ac.uk/ena/browser/view/PRJEB48874 and https://www.ebi.ac.uk/ena/browser/view/PRJEB50489. Accession codes for individual samples are provided in Supplementary Data 1. Species-level phylogenies are available in Supplementary Software 1, geographic and ecological characteristics of different species are available in Supplementary Data 4. Source data are provided with the paper.

Field-specific reporting

Please select the one below that is the best fit for your research. If you are not sure, read the appropriate sections before making your selection.

☐ Life sciences ☐ Behavioural & social sciences ☒ Ecological, evolutionary & environmental sciences

For a reference copy of the document with all sections, see nature.com/documents/nr-reporting-summary-flat.pdf

Ecological, evolutionary & environmental sciences study design

All studies must disclose on these points even when the disclosure is negative.

Study description

The aim of the study was to construct phylogenies for representative sample of mountain plant lineages that diversified in the European mountain system, using primary genomic data. The obtained phylogenies were used for exploring diversification dynamics and evolutionary assembly across ecological and geographic space.

Research sample

We aimed at acquiring a representative sample of plant lineages that significantly diversified in the European mountain system. The study lineages were selected based on following formal criteria:
- they contain more than 20 species in total, i.e. these are plant lineages that significantly diversified
- they contain at least 10 species inhabiting alpine and nival elevational belts in the European Alps, i.e. these are mountain plant lineages
- they were recently subject to taxonomic or phylogenetic revision suggesting that Europe is their center of diversity, i.e. these are European plant lineages
- they are eudicots, they do not follow derived life strategies as is myco-heterotrophy, parasitism or carnivory, i.e. we excluded lineages that fulfill previous criteria but were outliers in terms of life strategy

These criteria were met in 6 plant lineages (Androsace sect. Aretia, Campanula sect. Heterophylla, Gentiana sections Gentiana, Ciminalis and Calanthianae, Phyteuma, Primula sect. Auriculata and Saxifraga sect. Saxifraga), all of which were used in this study.

The material for genomic analysis of individual species from these lineages was sampled irrespective of individual age, as this variable could not influence results of genomic analyses. All species in the 6 lineages are hermaphroditic, so our sampling also did not follow any gender criteria. For taxonomic information and source of individual samples, please refer to Supplementary Data 1 and 2.

Sampling strategy

We aimed on sampling maximum of ingroup species from the focal plant lineages, and also their closest relatives as the outgroups. The sample size at the level of lineages was thus determined by the existence of suitable lineages in the European mountains system, and at the level of individual species and samples by a maximum labor intensity we were able to reach. We verified that this research sample is sufficient for questions we asked by a series of sensitivity and identifiability analyses (Supplementary Methods 2, 3 and 4).

Data collection

The data were collected by a consortium of research institutions and nature protection agencies, see Supplementary Note. Majority of samples for genomic analyses was taken in a form of living tissue of wild growing plants and quickly desiccated by insertion to silicagel, but several samples for genomic analyses were taken only in a form of herbarium. Information about sampling of individual accessions, including geographic coordinates, collector identity and silicagel/herbarium sampling procedure, is provided in Supplementary Data 1 and 2.

Timing and spatial scale

Data sampling took place in vegetation seasons of years 2008-2019. The collection dates of individual samples are provided in Supplementary Data 1. Timing of sampling could not influence the gathered genomic data in any sense.

The study relates to a geological interval between present and 40 Ma BP.

The spatial extent of the study is the European mountain system, i.e. all mountain ranges on the European continent west from 35 meridian. The spatial grain cannot be defined for most analyses, as they were spatially implicit and based on point collection data. Spatial analyses of sister species used two sizes of grain: Coarse grain, where operational geographic units were major European mountain regions (cca 300000-600000 km2); and fine grain, where operational geographic units were the smallest administrative for which we could attribute the occurrence information, considerably varying in size (cca 200-40000 km2).

Data exclusions	All unsampled ingroup species are reported in Supplementary Data 4. Construction of species-level phylogenies is described in Methods and Supplementary Methods 6.
Reproducibility	33 ingroup species were sampled and sequenced by two or more individuals for control purposes. In all the cases, these duplicated samples were closely related in final tree topologies, forming monophyletic or paraphyletic clades. Please refer to Supplementary Data 1 and 2, Supplementary Methods 6 and Methods for details.
Randomization	The study does not contain any experiment requiring group assignments of individual measurements or randomization.
Blinding	We did not use any blinding procedure in our study. The knowledge of sample identity in principle could not influence the outcome of data acquisition procedures such as collection in field, DNA extraction and sequencing.

Did the study involve field work? [x] Yes [ ] No

Field work, collection and transport

Field conditions	The sampling took place across the whole European mountain system, in areas with mean annual temperature between -10 and +20 °C, and mean annual rainfall between 200 and 4000 mm. The actual conditions during the 10 years campaign also varied considerably, but but we avoided sampling in situations where microsite temperature around sampled individual was below 0°C. Importantly, the fieldwork conditions could influence the collectors ability to find an individual suitable for sampling, but could not influence the collected genomic data.
Location	Collection sites across all the European mountain system, geographic coordinates and topographic descriptions of sites for individual accessions are provided in Supplementary Data 1.
Access & import/export	The habitats in which we performed the sampling were accessed on foot, in some cases with usage of mountaineering techniques. All the access activities were performed in publicly accessible areas and with respect to local regulations. Samples were collected, processed and stored in Europe, and their transportation did not violate any local regulations. Sampling of protected species in the Alps and Pyrenees was performed in collaboration with local nature protection institutions involved in the PhyloAlps consortium (National Botanical Conservatory Mediterranean, National Botanical Conservatory Alps, Ecrins National Park, Vanoise National Park, Mercantour National Park), along with collections permits for protected taxa (PN Mercantour, 2016-724, 29/06/2016; DDT Savoie, 2017-985, 20/07/2017; DDT Hautes-Alpes, 2017-07-21-001, 21/07/2017; DDT Haute-Savoie, 2017-1423, 21/07/2017; DDT Isère, 2017-07-20-003, 20/07/2017; DDT Alpes-Maritimes, 2017-07-18, 18/07/2017). Sampling of protected species in the Carpathians was performed in collaboration with Tatra National Park. Sampling of protected species in the Balkans was covered by permission from Croatian Ministry of Environment (no. 514-07-1-1-1-16-4, 30/06/2016).
Disturbance	Minimum amount of living plant material allowing extraction of DNA was collected for protected and other potentially vulnerable species. The cases of extremely small populations were specifically consulted with local authorities of nature protection (see above).

Reporting for specific materials, systems and methods

We require information from authors about some types of materials, experimental systems and methods used in many studies. Here, indicate whether each material, system or method listed is relevant to your study. If you are not sure if a list item applies to your research, read the appropriate section before selecting a response.

Materials & experimental systems

n/a	Involved in the study
[x]	[ ] Antibodies
[x]	[ ] Eukaryotic cell lines
[x]	[ ] Palaeontology and archaeology
[x]	[ ] Animals and other organisms
[x]	[ ] Human research participants
[x]	[ ] Clinical data
[x]	[ ] Dual use research of concern

Methods

n/a	Involved in the study
[x]	[ ] ChIP-seq
[x]	[ ] Flow cytometry
[x]	[ ] MRI-based neuroimaging
